# Understanding Health Communication Through Google Trends and News Coverage for COVID-19: Multinational Study in Eight Countries

**DOI:** 10.2196/26644

**Published:** 2021-12-21

**Authors:** Wai-kit Ming, Fengqiu Huang, Qiuyi Chen, Beiting Liang, Aoao Jiao, Taoran Liu, Huailiang Wu, Babatunde Akinwunmi, Jia Li, Guan Liu, Casper J P Zhang, Jian Huang, Qian Liu

**Affiliations:** 1 Department of Infectious Diseases and Public Health Jockey Club College of Veterinary Medicine and Life Sciences City University of Hong Kong Hong Kong Hong Kong; 2 Department of Public Health and Preventive Medicine School of Medicine Jinan University Guangzhou China; 3 School of Journalism and Communication National Media Experimental Teaching Demonstration Center Jinan University Guangzhou China; 4 College of Economics Jinan University Guangzhou China; 5 College of Economic and Management Nanjing University of Aeronautics and Astronautics Nanjing China; 6 International School Jinan University Guangzhou China; 7 Center for Genomic Medicine Massachusetts General Hospital Boston Armenia; 8 Faculty of Computer Science Jinan University Guangzhou China; 9 School of Public Health The University of Hong Kong Hong Kong Hong Kong; 10 MRC Centre for Environment and Health, Department of Epidemiology and Biostatistics School of Public Health, St Mary’s Campus Imperial College London London United Kingdom; 11 Department of Communication University at Albany, State University of New York Albany, NY United States

**Keywords:** COVID-19, Google Trends, search peaks, news coverage, public concerns

## Abstract

**Background:**

Due to the COVID-19 pandemic, health information related to COVID-19 has spread across news media worldwide. Google is among the most used internet search engines, and the Google Trends tool can reflect how the public seeks COVID-19–related health information during the pandemic.

**Objective:**

The aim of this study was to understand health communication through Google Trends and news coverage and to explore their relationship with prevention and control of COVID-19 at the early epidemic stage.

**Methods:**

To achieve the study objectives, we analyzed the public’s information-seeking behaviors on Google and news media coverage on COVID-19. We collected data on COVID-19 news coverage and Google search queries from eight countries (ie, the United States, the United Kingdom, Canada, Singapore, Ireland, Australia, South Africa, and New Zealand) between January 1 and April 29, 2020. We depicted the characteristics of the COVID-19 news coverage trends over time, as well as the search query trends for the topics of COVID-19–related “diseases,” “treatments and medical resources,” “symptoms and signs,” and “public measures.” The search query trends provided the relative search volume (RSV) as an indicator to represent the popularity of a specific search term in a specific geographic area over time. Also, time-lag correlation analysis was used to further explore the relationship between search terms trends and the number of new daily cases, as well as the relationship between search terms trends and news coverage.

**Results:**

Across all search trends in eight countries, almost all search peaks appeared between March and April 2020, and declined in April 2020. Regarding COVID-19–related “diseases,” in most countries, the RSV of the term “coronavirus” increased earlier than that of “covid-19”; however, around April 2020, the search volume of the term “covid-19” surpassed that of “coronavirus.” Regarding the topic “treatments and medical resources,” the most and least searched terms were “mask” and “ventilator,” respectively. Regarding the topic “symptoms and signs,” “fever” and “cough” were the most searched terms. The RSV for the term “lockdown” was significantly higher than that for “social distancing” under the topic “public health measures.” In addition, when combining search trends with news coverage, there were three main patterns: (1) the pattern for Singapore, (2) the pattern for the United States, and (3) the pattern for the other countries. In the time-lag correlation analysis between the RSV for the topic “treatments and medical resources” and the number of new daily cases, the RSV for all countries except Singapore was positively correlated with new daily cases, with a maximum correlation of 0.8 for the United States. In addition, in the time-lag correlation analysis between the overall RSV for the topic “diseases” and the number of daily news items, the overall RSV was positively correlated with the number of daily news items, the maximum correlation coefficient was more than 0.8, and the search behavior occurred 0 to 17 days earlier than the news coverage.

**Conclusions:**

Our findings revealed public interest in masks, disease control, and public measures, and revealed the potential value of Google Trends in the face of the emergence of new infectious diseases. Also, Google Trends combined with news media can achieve more efficient health communication. Therefore, both news media and Google Trends can contribute to the early prevention and control of epidemics.

## Introduction

In late December 2019, a cluster of patients with pneumonia of unknown etiology was reported in Wuhan, China [1]. Soon after, a new type of coronavirus was identified as the pathogen causing this pneumonia [2], which was named COVID-19 by the World Health Organization (WHO) [3,4]. As the number of COVID-19 infections continued to increase, the WHO declared COVID-19 a pandemic on March 11, 2020 [5]. Globally, as of July 2020, there have been more than 10.3 million confirmed cases and more than half a million deaths in over 200 countries [6], which caused global supply chain disruptions during the COVID-19 pandemic [7]. Therefore, the prevention and control of the epidemic require a great deal of urgency.

Surveillance is an essential component of infectious disease control [8,9]. Nevertheless, traditional public health surveillance of epidemic diseases is based on government-implemented data gathering, resulting in data that can take years to become available [10]. Traditional laboratory monitoring is still used in most countries, but in recent years, some countries have tried to use internet search query data to assist traditional public health surveillance, such as Google Flu Trends (GFT) and Google Dengue Trends [11-14]. In the future, various types of internet data, such as search data, will offer more possibilities for better disease prevention and control [11,12]. Google Trends is one of the most popular open online tools for assessing data from public internet searches and has multiple advantages [11]. Specifically, it collects real-time data automatically, and provides quantitative and qualitative data applied to the informatics research of various communicable and noncommunicable diseases [13,15]. For example, Ginsberg et al [16] employed Google to track influenza-like illness in a population. Ocampo et al [17] were the first to use Google search queries in malaria surveillance. Glynn et al [18] assessed the relationship between breast cancer awareness campaigns and internet search activity from 2004 to 2009 using Google Trends. All of the above research drew similar conclusions: Google Trends can supplement traditional public health surveillance and help us to better understand public response and sentiment to the pandemic. Moreover, Google Trends can help reveal the need for health-related information [11,19].

In addition, news coverage of COVID-19 by mass media played an important role during the outbreak [20]. As a source of information, news coverage can provide important information to the public and, in turn, guide people to form positive, healthy behaviors or prevent the development of unhealthy behaviors. News coverage influences the behaviors of the public by both direct and indirect routes: news content can directly influence the behavior of the recipients or indirectly influence interpersonal discussion and transmission of coverage content [21,22]. For instance, the public’s online search behaviors for information about diseases increase during disease awareness months [18,23]. Moreover, some researchers have noted that internet search behaviors and news coverage were relevant to traditional data monitoring, and the latter appeared to promote internet searches for health topics [24,25]. In the area of public health [26], when there is an emerging pandemic, news media as a tool can inform the public about prevention and control strategies. On the other hand, news media can also have a negative side. For example, news coverage might not be based on expert assessments and may hold relatively independent views. Also, news coverage might cause public panic. Although newsworthiness is complex, analyzing internet data can help improve the effectiveness of public communication [19]. In other words, news coverage plays an important role in health communication. Hence, acquiring available online data, including internet search query data and social media information, can provide novel insights for the prevention and control of COVID-19 [27].

To date, only a few studies have focused on internet search data combined with news coverage data. This study, therefore, used Google query data, news coverage data, and new COVID-19 case data to understand health communication during the early stage of this epidemic.

## Methods

### Overview

In this study, we collected data from Google Trends, news coverage, and new COVID-19–related daily cases from January 1 to April 29, 2020 (120 days), which is considered the early period of the epidemic in eight countries: the United States, the United Kingdom, Canada, Singapore, Ireland, Australia, South Africa, and New Zealand. We then described different Google Trends search queries and news coverage trends in different countries to understand the situation of health communication, and we explored the connection between the above and the prevention and control of COVID-19 at the early epidemic stage.

### Data Collection

#### Google Query Data

Google Trends is one of the most popular online tools used to track internet hit search volumes. Users of Google Trends [28] can obtain the search trend data of terms [8]. Google Trends provides a relative search volume (RSV) to depict the popularity of a specific search term in a specific geographic area over a period of time. The value of RSV ranges from 0 to 100. A value of 0 means there was not enough data for this term, and a value of 100 represents the peak popularity for the term [10,29].

Based on a previous study [20], symptoms, treatments and medical resources, measures, and the virus itself were the major topics covered by online media during the early period of the COVID-19 pandemic. Therefore, we selected “diseases,” “treatments and medical resources,” “symptoms and signs,” and “public measures” as search topics, and we used their terms as search terms. Also, due to the limited language of Google Trends, only English-speaking countries were included in this study [30]. According to population size, we selected eight English-speaking countries for the study: the United States, the United Kingdom, Canada, Singapore, Ireland, Australia, South Africa, and New Zealand. RSV data for the above topics in these eight countries, between January 1 and April 29, 2020, were collected and then exported into CSV files. The topics and their query terms are shown in Table 1.

**Table 1:** Query topics and search terms related to COVID-19.

Query topic	Search terms
Diseases	“coronavirus,” “covid-19,” and “pneumonia”
Treatments and medical resources	“ventilator,” “vaccine,” and “mask”
Symptoms and signs	“fever,” “cough,” “shortness of breath,” and “tiredness”
Public measures	“quarantine,” “lockdown,” and “social distancing”

#### News Coverage Data

Meltwater is a platform that provides real-time monitoring of domestic and overseas news, and covers more than 300,000 online websites, news clients, and other news media [31]. With wide geographical coverage, Meltwater provides rich news data from different countries. To compare and analyze the news media coverage on COVID-19, we selected news media from eight countries (ie, the United States, the United Kingdom, Canada, Singapore, Ireland, Australia, South Africa, and New Zealand) and searched the news coverage from January 1 to April 29, 2020, with “covid-19” or “coronavirus” as the keywords.

#### New Case Data

The number of new daily cases of COVID-19 was obtained from the WHO with surveillance data [32].

### Analytical Framework

First, we used line graphs to show search trends for different topics in eight countries and attached the epidemic curves of new COVID-19 cases. We then assessed the most popular terms at the country level by comparing their search peaks to determine the characteristics of various terms in different countries. We then explored the reason for trend fluctuation of search query terms and the fluctuation impact on the prevention and control of COVID-19. Additionally, in Google Trends, the plus sign (+) has the function of “OR” and can be used to connect multiple terms to form an overall term [33]. Thus, we used “+” to integrate multiple terms in different topics into the overall term of the topic, and its RSV represents the overall RSV of the topic. For example, we used the RSV of “coronavirus + covid-19 + pneumonia” to represent the overall RSV of “diseases.”

Second, we used the neighborhood average method to smooth the news coverage data [34,35]. Then we used line charts to show news coverage longitudinal trends and identified the similarities and differences of news coverage between eight countries. Furthermore, to further discuss the relationship between news coverage and internet search queries, as well as the relationship between search queries and daily news, we summed the overall RSVs of the four topics to obtain the total RSV and attached it to the line chart along with the epidemic curve of new daily cases to more intuitively observe the changes of the three in the different countries. Moreover, we conducted time-lag correlation analysis between the overall RSVs of search queries for different topics and the number of new COVID-19 cases each day, as well as between the overall RSVs of search queries for different topics and the number of daily news items. The cross-correlation function of the “tseries” package from R software (version 4.0.5; The R Foundation) was used to compute time-lag correlations. In the analysis, a time lag between –17 and +17 days was used, and the Pearson correlation coefficient was used as the correlation measure.

Finally, the interrupted time series analysis was used to evaluate the impact of the appearance of the first COVID-19 case on the four search terms of the topic “symptoms and signs.” Taking the date of the first COVID-19 case as the change point, we used the generalized least squares estimator to fit the segmented linear regression model to evaluate the change in the level and slope of the RSV after the first case was discovered. Also, the residual autocorrelation was tested using the Durbin-Watson test. All hypothesis tests used a significance level (α) of .05.

## Results

**Figure 1:**
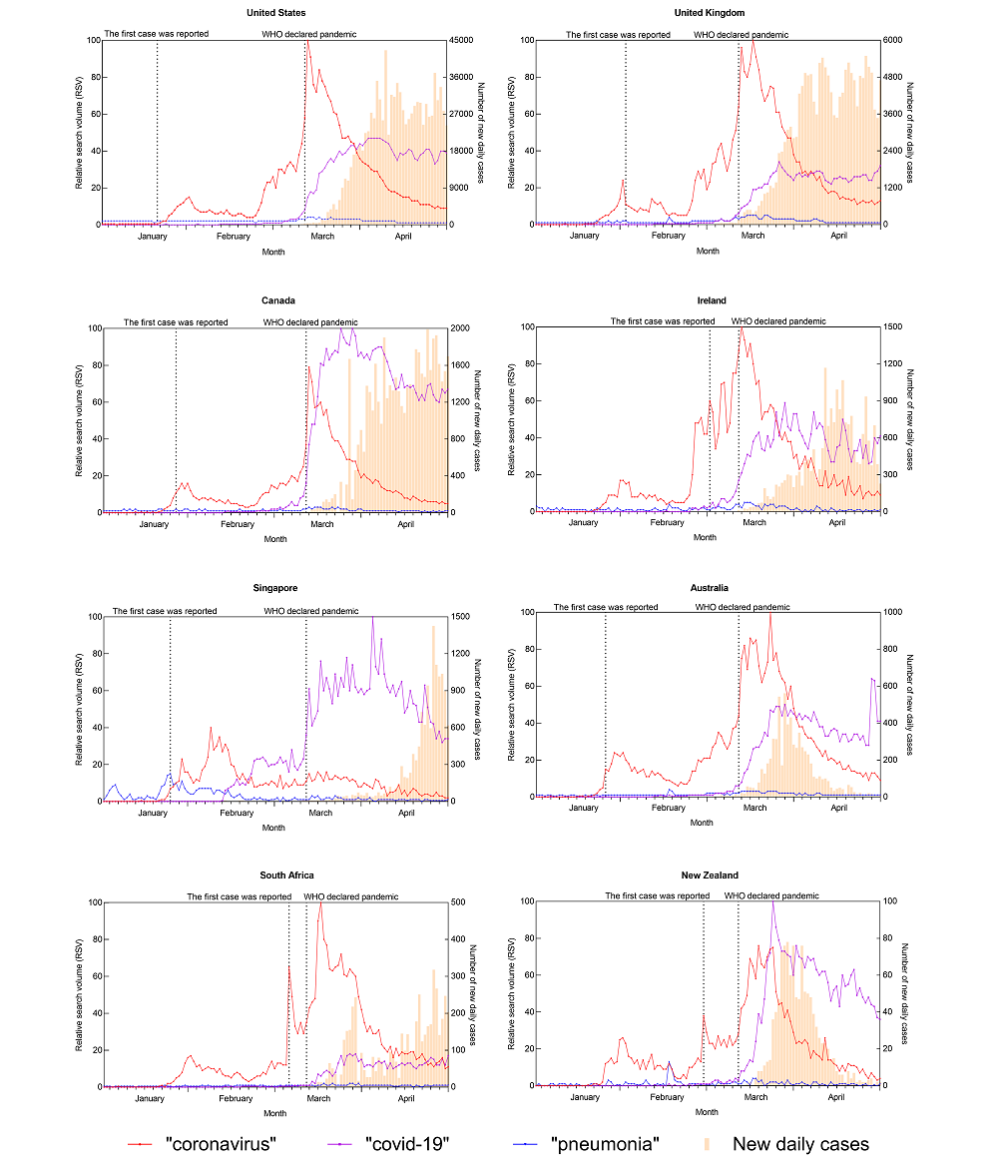
Search query trend of the “diseases” topic and the trend of new daily COVID-19 cases for eight countries from January 1 to April 29, 2020. WHO: World Health Organization.

**Figure 2:**
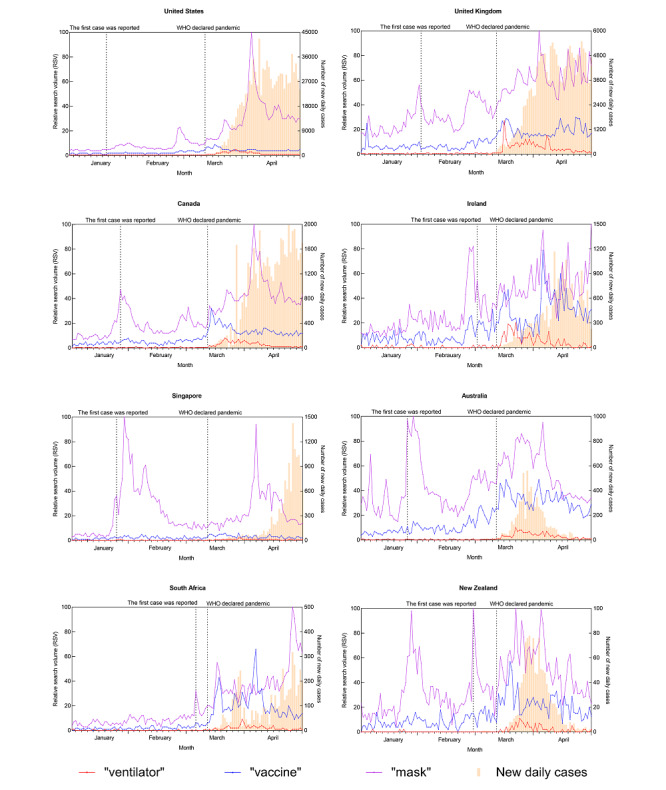
Search query trend of the “treatments and medical resources” topic and the trend of new daily COVID-19 cases for eight countries from January 1 to April 29, 2020. WHO: World Health Organization.

**Figure 3:**
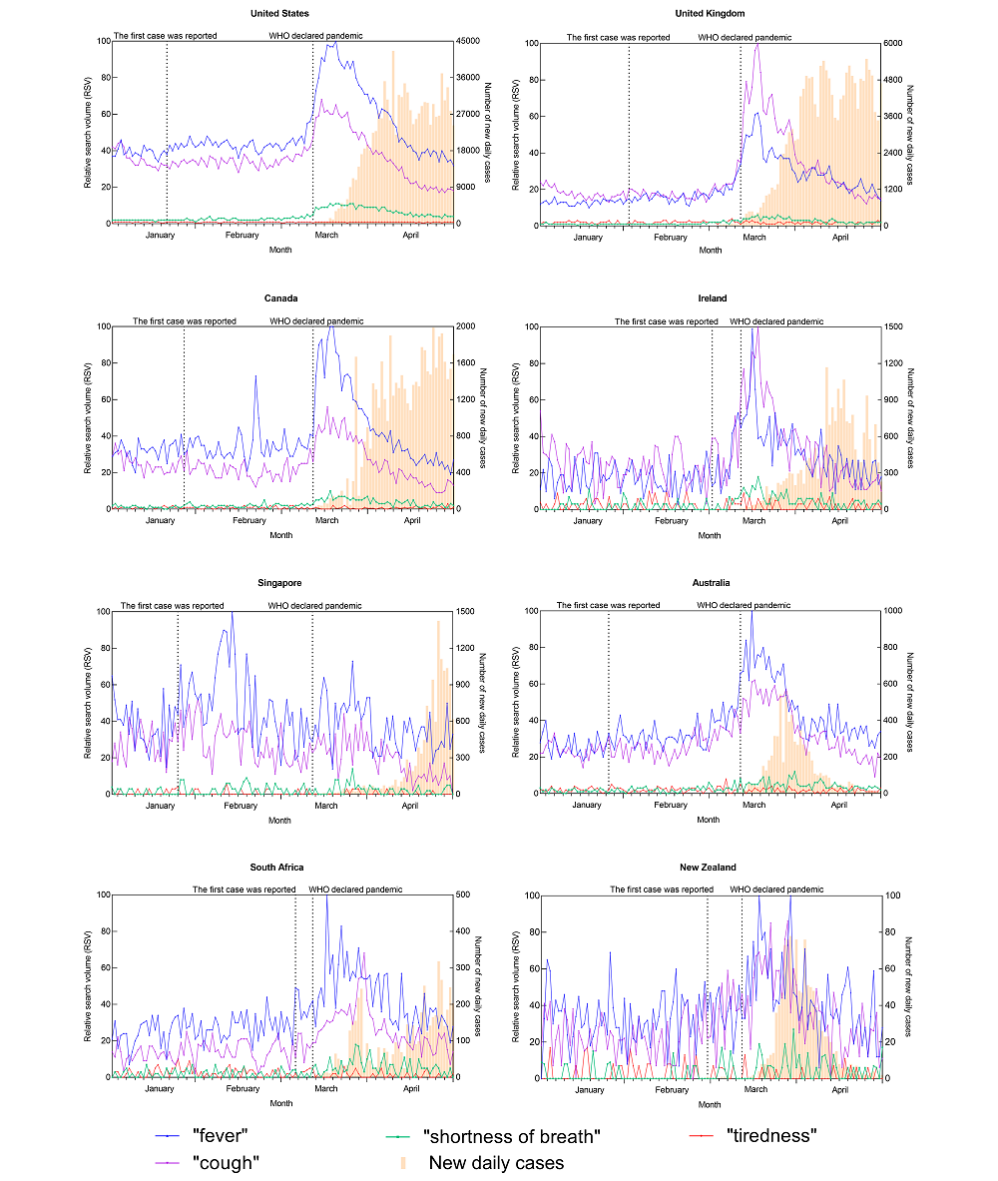
Search query trend of the “symptoms and signs” topic and the trend of new daily COVID-19 cases for eight countries from January 1 to April 29, 2020. WHO: World Health Organization.

**Figure 4:**
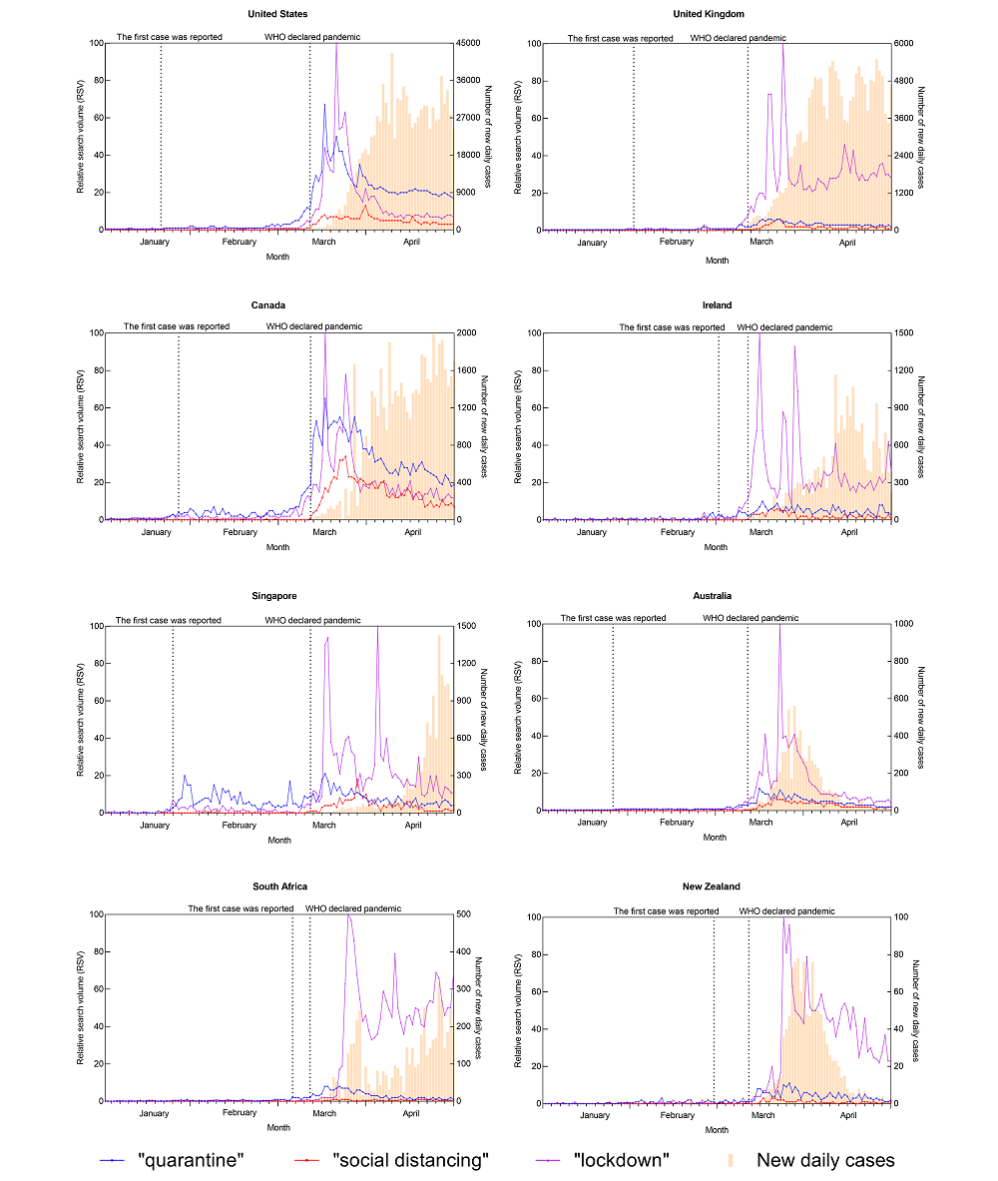
Search query trend of the “public measures” topic and the trend of new daily COVID-19 cases for eight countries from January 1 to April 29, 2020. WHO: World Health Organization.

Figures 1 to 4 depict the trends of a specific query topic by its associated query terms, accompanied by new daily cases in the eight countries studied.

For the topic “diseases,” we used the search terms “coronavirus,” “covid-19,” and “pneumonia” (Figure 1). Regarding the term “coronavirus,” its RSV increased around January 20, 2020, with a small peak at the end of January 2020. Except for Singapore, the RSV of “coronavirus” in other countries all formed an obvious peak in mid to late March 2020. Regarding the term “covid-19,” its RSV began to increase on February 11, 2020, and generated the top search peak from late March to early April 2020; around April 2020, the RSV value of this term surpassed that of “coronavirus.” Compared to these two terms, the trend for “pneumonia” fluctuated very little between January and April 2020.

Figure 2 shows the trends of the topic “treatments and medical resources,” including the query terms “ventilator,” “vaccine,” and “mask.” The term “mask” was the most searched term, followed by “vaccine” and “ventilator.” Regarding the term “mask,” there was one main search peak that occurred in April 2020 for all eight countries despite multiple spikes found in specific countries (ie, Singapore, Ireland, Australia, and New Zealand). Regarding the term “vaccine,” its RSV for most countries rose starting in March and generated several small spikes near mid-March 2020.

Figure 3 shows the trends for the topic “symptoms and signs” related to COVID-19. Among its query terms, “fever” was the most searched term, followed by “cough,” “shortness of breath,” and “tiredness.” Regarding the terms “fever” and “cough,” their top search peaks were formed around mid-March 2020 for all countries except Singapore, slightly earlier than the peak of new daily cases. In Singapore, the search peaks of “fever” and “cough” appeared between late January and mid-February 2020.

Figure 4 shows the trend for the topic “public measures,” using the query terms “quarantine,” “social distancing,” and “lockdown” during this study period. The RSV of “lockdown” was the highest, followed by “quarantine” and “social distancing.” For all these terms, their RSVs were very low before March 2020, and the RSVs of “quarantine” and “lockdown” increased and formed search peaks after mid-March 2020.

**Figure 5:**
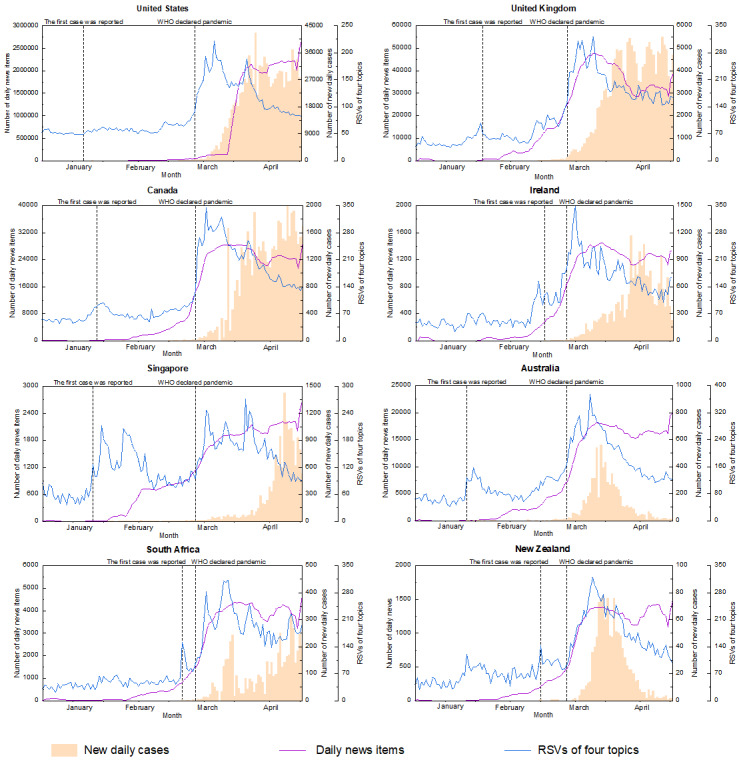
News coverage trends, new daily cases, and total relative search volumes (RSVs) of four topics for eight countries from January 1 to April 29, 2020. WHO: World Health Organization.

News coverage trends related to COVID-19 are shown in Figure 5. According to the neighborhood average method, we set 7 days as a base period to smooth the number of news coverage items. With the United States as an example, *y*_1_, *y*_2_,..., *y*_n_ were the true number of news coverage items from January 1 to April 29, 2020, where n=120. Therefore, the fitted value of news reports *S_t_* could be obtained by *S_t_* = (*y_t–3_ + y_t–2_ + y_t–1_ + y_t_ +y_t+1_ + y_t+2_ + y_t+3_*) / 7, where *y_t–3_*, *y_t–2_*, *y_t–1_* represents the true number of news coverage items about 3 days, 2 days, and 1 day before day *t*, and *y_t+3_*, *y_t+2_*, *y_t+1_* represents the true number of news coverage items about 3 days, 2 days, and 1 day after day *t*, where *t*=4,..., 117. Across eight countries, the number of news reports remained low before February 2020. From the end of January, the news report number gradually increased until the end of March 2020 and remained stable afterward. This trend was consistently observed in all countries, except the United States. In contrast, the coverage in the United States soared from around March 29, 2020, far outpacing that in any other country by nearly 300 times. Also, when comparing the trends of the total RSVs and news coverage, we identified three main patterns across the eight countries, which we have termed Singapore, the United States, and other country patterns. In Singapore, the trends of total RSVs formed two major peaks between late January and mid-February and between mid-March and early April, respectively, and the number of news reports increased gradually to a relatively high level starting from the end of January 2020. In the United States, as the total RSVs reached a peak around mid to late March 2020, the total RSVs began to decline, while the amount of low-level news coverage suddenly increased to a relatively high level at the end of March 2020. In other countries, the total RSVs and the number of news coverage items spiked in mid-March, but the growth of total RSVs occurred slightly earlier than that of news coverage items. Across all patterns, the total RSVs gradually dropped to the baseline level after the peaks from mid-March to early April, while the news coverage items remained at a higher level.

**Figure 6:**
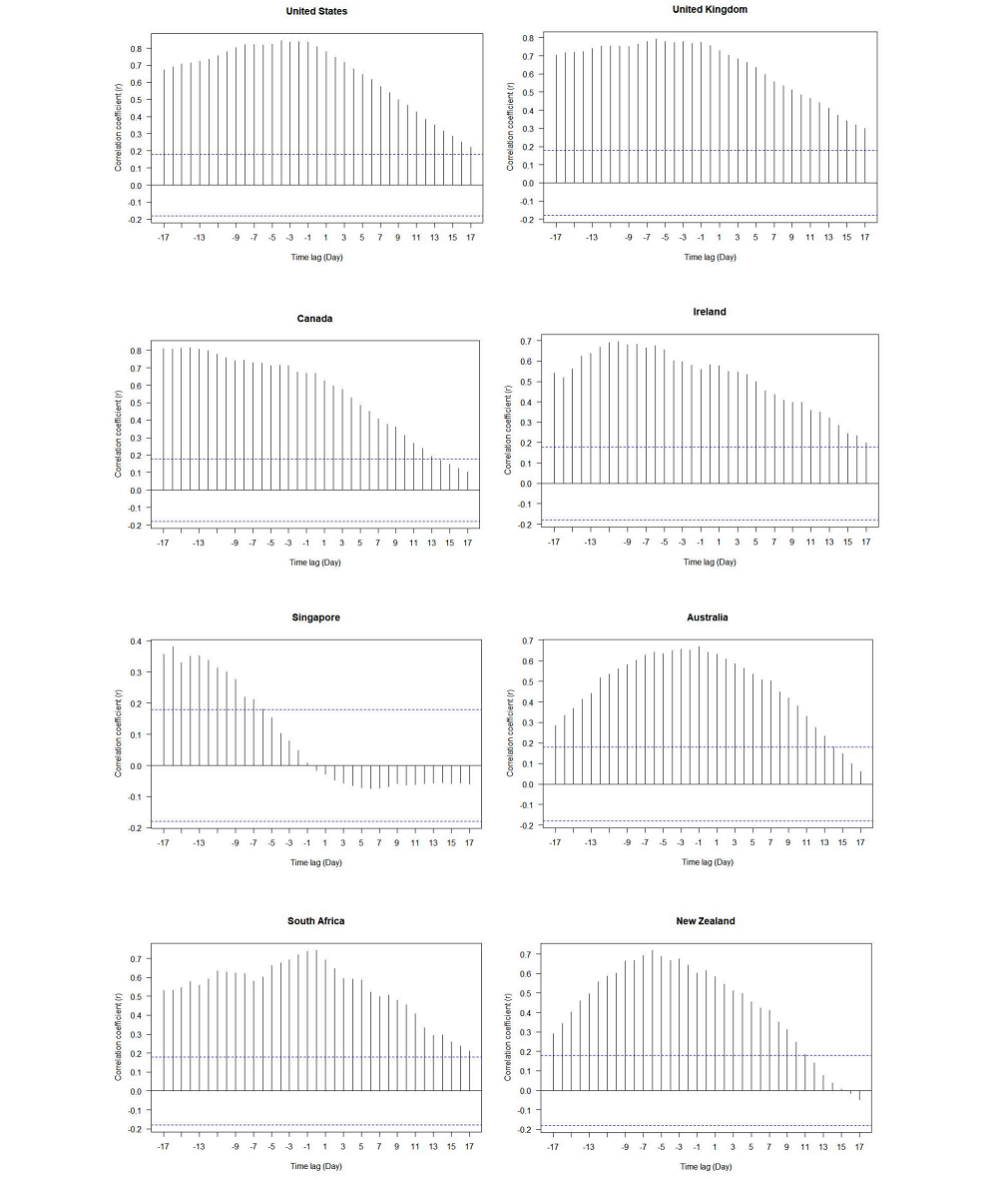
Time-lag correlations of the overall relative search volume (RSV) for the “treatments and medical resources” topic and new daily cases for eight countries from January 1 to April 29, 2020. The area between the two dotted blue lines is the 95% CI of the white noise. If the correlation coefficient of the time lag z days falls between the two blue dotted lines, we could believe that the new daily cases are not related to the overall RSV of “treatments and medical resources” within the lag (pre) z days when the maximum number of new daily cases was reported, with 95% confidence level.

Figure 6 shows the time-lag correlation between the overall RSV for the topic “treatments and medical resources” and the new daily cases. With the exception of Singapore, there was a positive correlation between the overall RSV for the “treatments and medical resources” topic and the new daily cases in all countries, with the highest correlation being 0.8 for the United States. Also, we divided the eight countries into three categories: (1) Singapore; (2) the United States, the United Kingdom, Canada, South Africa, and Ireland; and (3) Australia and New Zealand. In Singapore, the overall RSV for the “treatments and medical resources” topic gradually decreased within 17 days before the peak of new daily cases of COVID-19; after forming the peak of new cases, there was a clear negative correlation. In the second category of countries (ie, the United States, the United Kingdom, Canada, South Africa, and Ireland), the overall RSV for the “treatments and medical resources” topic was maintained at a high level for about 17 days before the peak of new daily cases was formed, and then decreased gradually; the correlation remained above 0.2. In other words, the correlation between the overall RSVs of these countries and the new daily cases was maintained at a medium to high level during the time lag of –17 to 17 days. In the third category of countries (ie, Australia and New Zealand), about 1 day and 6 days before forming the peak of new daily infections, the overall RSV for the “treatments and medical resources” topic reached the highest levels, with the maximum correlations being close to 0.8 and 0.7. The time-lag correlation between –17 and 17 days showed a high curve trend in the middle and was low on both sides.

**Figure 7:**
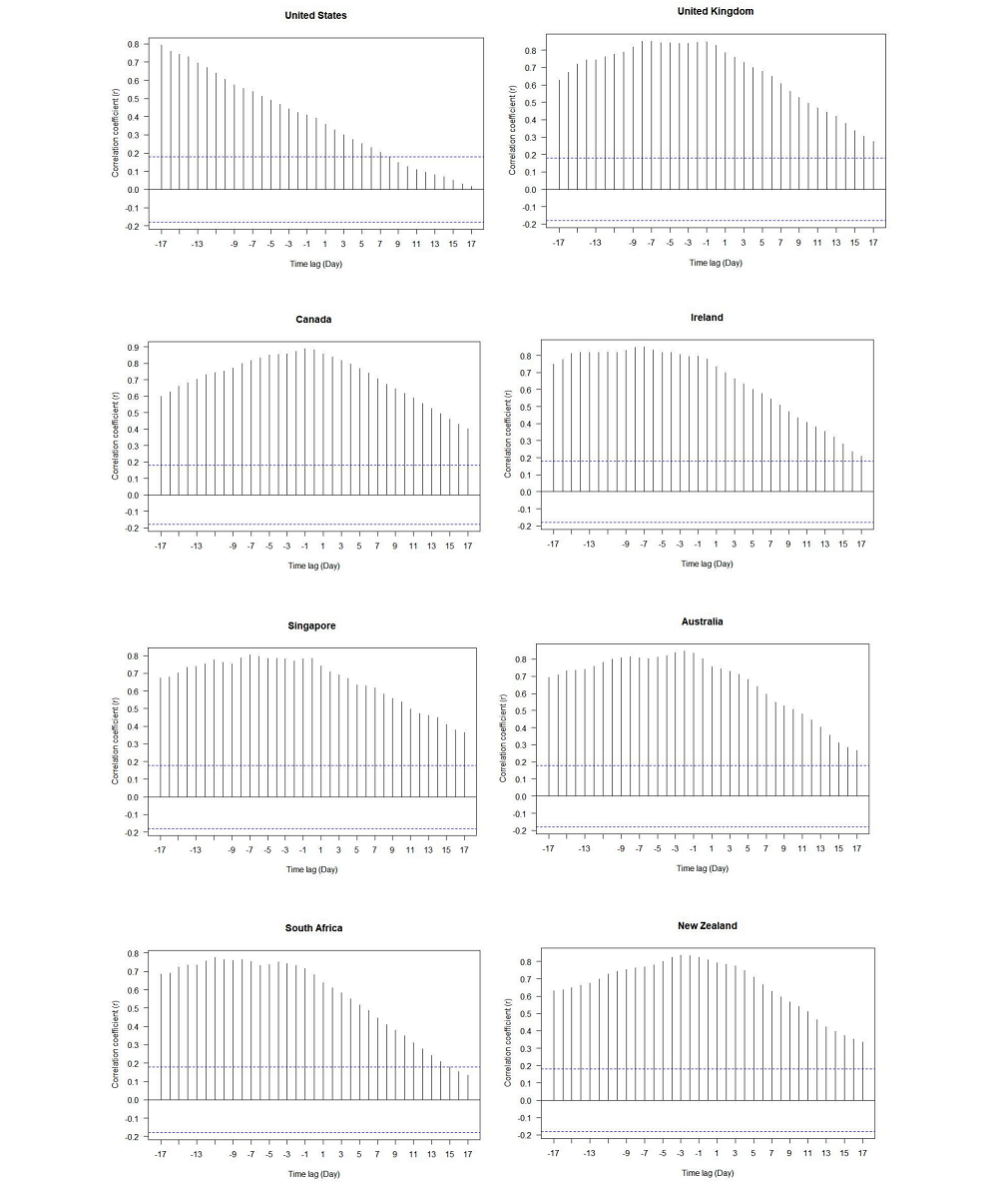
Time-lag correlations of the overall relative search volume (RSV) for the “diseases” topic and daily news items for eight countries from January 1 to April 29, 2020. The area between the two dotted blue lines is the 95% CI of the white noise. If the correlation coefficient of the time lag z days falls between the two blue dotted lines, we could believe that the daily news items are not related to the overall RSV of "diseases" within the lag (pre) z days when the amount of daily news coverage reached the maximum, with 95% confidence level.

Figure 7 shows that there was a positive correlation between the overall RSV for the topic “diseases” and the number of daily news items in eight countries, with the highest correlation coefficient exceeding 0.8; this indicated that as the number of search queries on the topic of “diseases” increased, the number of daily news items related to COVID-19 also showed an increasing trend. We divided the eight countries into two categories. The first category included only the United States; its maximum correlation appeared in the 17 days before the largest number of daily news reports, and then the correlation gradually decreased within the time lag from –17 to 17 days and showed an obvious negative linear trend. That is, the public’s interest in the topic of “diseases” reached its peak 17 days before the peak of news coverage and then gradually decreased over time. The second category included the United Kingdom, Canada, Ireland, Singapore, Australia, South Africa, and New Zealand. During the 17 days before the largest amount of daily news, public interest in the topic of “diseases” remained high. Most of these countries reached the highest level of public interest in “diseases” in about 1 day before the largest amount of daily news; the maximum correlation was close to 0.8. However, within 17 days after the largest amount of daily news, the public gradually lost interest, but most of the correlations remained above 0.2; that is, the correlations maintained a moderate level.

Figures S1 to S3 in Multimedia Appendix 1 show the results of the time-lag analysis between the overall RSVs for the topics “diseases,” “symptoms and signs,” and “public measures” and the number of new daily cases. Figures S4 to S6 in Multimedia Appendix 1 show the results of the time-lag analysis between the overall RSVs of the topics “treatments and medical resources,” “symptoms and signs,” and “public measures” and the number of daily news items. Table S1 in Multimedia Appendix 1 reports the effect of the first COVID-19 case on the RSVs of the search terms for the topic “symptoms and signs.”

## Discussion

### Principal Findings

Regarding the search trends of the topic “diseases,” all of the search peaks were earlier than new cases of COVID-19; this was similar to other studies [25,36,37]. When “coronavirus” was used as a search term, this term caused a spike of interest in all countries around January 20, 2020. On that day, the Chinese authorities announced that the virus was contagious, and the first case was found in the United States, which may have prompted the public to quickly recognize the threat and raised public interest. The term “covid-19” was first published by the WHO on February 11, 2020. Since then, its search volume has gradually increased and surpassed the terms “coronavirus” and “pneumonia” to become the main search term for this pandemic. The above findings showed that there were changes in public interest in external events related to the COVID-19 outbreak, indicating that Google Trends had the potential to be used as a tool to monitor public reaction and emotion regarding threatening events [38].

Regarding the search trends of the topic “treatments and medical resources,” the public was the least interested in the term “ventilator,” despite this being an important piece of medical equipment for the treatment of COVID-19 patients, and there was a shortage of ventilators in some countries or regions during the epidemic, such as New York City [39]. However, the majority of healthy persons were more concerned with masks than ventilators. Furthermore, wearing masks is an important means of preventing infection and plays a crucial role in curbing the COVID-19 epidemic [40]. In the situation of mask shortages [41], the public’s interest in the term “mask” showed great fluctuation; although the reasons for the change in search behaviors were complex, it largely reflected public concern about the shortage of masks to some extent. In addition to masks, vaccination is an important way to end the COVID-19 pandemic [4]; as such, rising public concern reflected by the term “vaccine” was observed in our study, which was consistent with the findings in a previous study by Paguio et al [38]. In the face of the rapid spread of COVID-19 and the lack of effective vaccines, the public has paid much attention to vaccine research, in part reflected by the panic related to the urgent public need for COVID-19 vaccines, which might also indicate hope in ending the current pandemic [38].

Furthermore, in the time-lag correlation analysis, there was a positive correlation between the overall RSV for the topic “treatments and medical resources” and new daily cases for all countries except Singapore, where the maximum correlation coefficient exceeded 0.8 for the United States. In addition, the overall RSV peak for the topic “treatments and medical resources” occurred 0 to 17 days earlier than the peak for new daily cases. The positive correlation coefficient showed that as the search volume increased in this study, the number of new daily cases also showed increasing trends. These results were similar to those from other studies [25,42,43]; therefore, Google Trends has the potential to become a useful tool for disease prevention and control. Moreover, Ali et al [44] found that by observing Google Trends, the public’s interest in telemedicine continued to increase. However, in most countries and regions, the health care system’s digital equipment was unable to meet growing public demand, which reminded relevant stakeholders to incorporate telemedicine into the health care system to combat pandemics. In a study by Nikolopoulos et al [7], the researchers also used Google Trends data and simulated government policies to model and successfully predict the excessive demand for products and services during the pandemic. The results showed that Google Trends data could identify the dynamic process of prediction and supply chain management directions in order to assist decision makers in making many key decisions on supply chain and disease prevention strategies. Therefore, Google Trends could be used to capture the public’s early concerns or needs in order to identify fluctuations in public demands [7]. During a public health crisis, the RSV increase for specific topics or terms could be regarded as public demands or needs; we could translate these public demands into practice to formulate reasonable countermeasures to respond quickly [45]. For example, Google Trends could provide an opportunity to formulate production plans to avoid supply chain disruptions and ensure reasonable allocation of resources. Specifically, the government could arrange special fiscal budgets in advance to cover expenses related to public health emergencies and their associated impacts, such as subsidies for companies that produce masks and ventilators [45]. However, we still need more research to provide much more evidence about the predictive value in supporting decision-making policies.

For risk surveillance of emerging infectious diseases, syndromic surveillance might detect health threats faster than traditional surveillance systems, thus making timely public health action more likely [46]. Recently, Google Trends data have been applied to syndromic surveillance: this is based on the principle that when patients have a certain symptom, they are likely to search for the description of this symptom on Google. When the RSV of one particular symptom is increasing, the syndromic monitors can be alerted after a series of extensive analyses [11]. In this study of “symptoms and signs” search trends, fever and cough were symptoms that the public was most concerned about in most countries, which have been reported as the most common symptoms of COVID-19 [47]. Meanwhile, the results of the time-lag correlation analysis showed that the search peaks for the “fever” and “cough” terms were 1 to 17 days earlier than the peak of new cases in each country, with the maximum correlation coefficient being close to 0.9 for Australia; this supports Google Trends data indicating that the above symptoms seemed to act as a warning function during the early epidemic period. Also, many researchers had used specific search data to accurately estimate the level of weekly influenza activity [16,48]. In other words, there might be a certain relationship between search query data and the number of new cases, which is likely to be useful for surveillance, prevention, and control of COVID-19. However, there has been debate about the usefulness of Google search query data for predicting pandemics; the cancellation of GFT suggests that the predictions by this tool might not be sufficiently accurate [49]. Generally, syndromic surveillance often cannot fully reflect the epidemic status of the disease and will be affected by other factors, such as news coverage and important events [36,50]. In other studies, media reports have been proven to be an important factor affecting search query interest [51]. In this study, the peak RSV was earlier than the peak number of news reports, and the trend of RSV was still positively correlated with the number of news reports (Figure S5 in Multimedia Appendix 1). Therefore, although the predictive value of Google Trends is questionable, future research studies might need to eliminate the influence of factors such as media reports.

For the prevention and control of infectious diseases, quarantine, social distancing, and lockdown are all public measures that are used to control the source of infection and block the route of transmission, which are extremely important for the prevention and control of COVID-19 [52]. Regarding the “public measures” topic, the search trend peak was formed in mid to late March, and the corresponding important event was that the lockdown policies of most countries were also released and implemented in mid to late March [51]. Similarly, from the results of the time-lag correlation analysis, the peak public interest in all countries except the United States was close to the peak number of news reports, but the peak of reporting on COVID-19–related news was slightly later than the peak of the public’s interest (Figure S6 in Multimedia Appendix 1). Moreover, the RSV of the term “lockdown” was significantly higher than that of the term “social distancing.” In addition to indicating that citizens in most countries were more interested in the term “lockdown,” it might be that the public was not clear about the meaning of the public measure of “lockdown.” The effectiveness of public measure interventions depends not only on strong policies but also on the correct cognition and compliance of the public measures. Thus, if the public lacked interest or understanding in public measures, this could jeopardize COVID-19 prevention and control [47,52,53]. Also, news media is an important tool for achieving good risk communication at the early stage of an infectious disease epidemic and for improving the control effect of policies or measures [26]. Therefore, before or at the initial stage of implementing new policies or measures, the government can use the news media to propagate policies and develop a good risk communication strategy to obtain high-quality health communication effects to better control the spread of COVID-19 [54].

When comparing search query trends with news coverage, the search query trends showed public interest, and the news reflected mass health communication. Also, the number of new cases was one indicator reflecting the severity of the epidemic and the level of prevention and control. Under the eight countries’ different cultural, political, and epidemic situations, there were three health communication patterns: (1) the pattern for Singapore, (2) the pattern for the United States, and (2) the pattern for the other countries. Regarding the pattern for Singapore, it was quite different from that of the other countries. The biggest difference was that the search query peaks appeared earlier than those of the other countries, indicating that Singaporeans were more concerned in the early period of the epidemic. Moreover, in Singapore, the results of the time-lag analysis between the “treatments and medical resources” topic, the “symptoms and signs” topic, the number of daily news items, and the number of new daily cases were also different from those of the other countries. The correlation was negative and low. Among them, the correlation between the Singaporean public’s search interest in “treatments and medical resources” and the number of daily news items was low (Figure S4 in Multimedia Appendix 1), indicating that at the early stage of the COVID-19 epidemic, the Singapore public’s early attention toward “treatments and medical resources” was less likely to be affected by the number of news reports, but was likely to be affected by other factors. Two main reasons could be used to explain the Singapore public’s interest. One was that Singapore, as a tourism hub, has frequent tourism-business exchanges with neighboring China. The other was that Singapore had learned hard lessons from SARS in 2003 [55], so it had taken various measures to control the spread of the virus early in the epidemic, such as temperature checks and health screening, public education, and quarantine. These measures potentially made the public aware of a new threat and relevant health information as soon as possible and, thus, improved the public’s sensitivity and vigilance to COVID-19 via health communications [56,57]. In other words, Singapore had done a good job of containment and prevention at the early stage. Similarly, the Singaporean public’s early interest in symptoms was likely affected by other factors or events, such as the first COVID-19 case (Table S1 in Multimedia Appendix 1), though the determination of the cause of RSV changes needs further analysis.

Regarding the patterns of the United States and other countries, the amount of news coverage in the United States was much higher than in other countries. The number of new cases was also far higher than in other countries. Therefore, to some extent, their level of news coverage related to COVID-19 was justifiable, but that might also be an illusion caused by the irregularity of the data collection methods. In general, news coverage in most countries was highly responsive to the COVID-19 epidemic in late March. In addition, the results of the time-lag correlation analysis between the number of daily news items and the overall RSV for the topic “diseases” also reflected the fact that news reports appeared later than search queries, with lag times ranging from 0 to 17 days. Moreover, the correlation between the two was relatively high and gradually decreased over time, indicating that in this study, the public’s interest in the COVID-19 outbreak occurred earlier than the appearance of news media reports. Based on Dutta-Bergman’s channel complementarity theory, Zillmann and Bryant’s selective exposure theory, and Rubin’s use and satisfaction theory, which assume that active audiences use different media channels to meet their needs [58], we may use these to explain the relationship between news coverage and search query trends. To be specific, in the uncertainty of this COVID-19 epidemic, there was initially little news coverage, indicating that the public was probably not sufficiently informed, so the public’s search volume was higher. As the news coverage increased, more information was available, and uncertainty decreased, as did the online search behavior of the public. However, the number of overall RSVs in the same period began to decline, which might be a kind of public desensitization for COVID-19, likely caused by continuous extensive news coverage [59-61]. That is, at the early stage of the COVID-19 epidemic, there was an increase in health information–seeking behaviors because the public lacked relevant information [42]. Therefore, in this case, Google Trends could reflect information needs and potentially provide appropriate window periods and locations for risk communication and health communication [42,62].

In the face of emerging infectious diseases, the public lacks relevant information, and timely and effective risk communication is necessary. News media is a key resource in shaping public awareness of risks and communicating relevant health information; it has great potential to become an effective partner in health communication, which could promote risk communication and the implementation of disease prevention and control strategies [26]. In this research, the public’s interest in different topics had different characteristics, and their interest was related to factors such as the development of the epidemic and media reports. This also reminded countries or public health departments that when communicating with the public, they should unite with the news media as soon as possible, pay close attention to changes in public interests by monitoring Google Trends search data and media reports, plan the nature and content of news items, and provide the information needed by the public in a more reasonable manner, in order to better prevent and control epidemics at their early stages, such as the COVID-19 epidemic [26,43,54]. However, the RSVs of the search terms from Google Trends are relative values and do not provide the exact values of the actual search volumes. As some search terms with higher search volumes appear, the change in trend of search terms may be underestimated [63]. As a result, it somewhat reduces the usability of Google Trends, though the linear trend of individual search terms does not change. However, in some studies, by collecting more data to analyze seasonal differences and long-term trends, we can further analyze whether there are changes in search terms and explore the meaning and reasons of these changes [17,38]. In addition, Google Trends has the characteristic of being available in real time, which can not only be used to monitor public emotions, reactions, and needs in real time, but can also be used to evaluate the effects of risk communication and public health interventions and the impact of major events or policies, among other factors. For example, interrupted time series analysis was used to assess the impact of celebrity suicides on search volumes, as well as the impact of tobacco control policies on search rates for smoking cessation information, in order to evaluate the effectiveness of the policy implementation [64,65]. In the internet era, with the popularity of mobile terminals, online searching is a two-way communication process, including sending search requests and receiving search results. Sending a search request reflects the public’s response to the severity and urgency of the risk and actual needs, and receiving search results provides feedback in response to the public’s views on their ability and effectiveness to manage or respond to risks [66]. Therefore, timely responses and exploration of data are very important, and Google Trends has the characteristic of real-time availability. In addition, Google Trends can also integrate more data sources, such as Twitter and Facebook, among others, so Google Trends data are still valuable [39,67-69].

### Limitations

Google Trends has its own limitations. For example, it is more applicable to study high-prevalence diseases in countries where the internet is popular [8] and when providing a relative versus exact value for search volume. Due to Google’s existing language limitations [23], we only studied the major English-speaking countries. Also, Google search data and news data might not be comprehensive enough and might not have included all of the search terms or topics related to COVID-19. For example, we did not include some important symptoms (eg, “loss of taste or smell”), and we omitted some similar terms such as “Wuhan virus.” In addition, “pneumonia” was not related only to COVID-19, but could also be related to influenza. Also, there was no one-to-one correspondence between news coverage data and search terms and topics. Therefore, further studies should apply detailed search terms and extract more news data to explore additional values.

### Conclusions

Through Google Trends, we identified the level of public interest for various aspects at the early stages of the COVID-19 epidemic, learned about public concern and neglect, and revealed the potential value of Google Trends in monitoring public response and demand, prediction, and other aspects in the face of the occurrence of emerging infectious diseases. In addition, news media as an essential source of information, combined with Google Trends, could achieve more effective health communication. Therefore, both news coverage and Google search trends could potentially contribute to the prevention and control of epidemics at the early epidemic stage.

## Appendix

Multimedia Appendix 1 Supplementary materials.

## Abbreviations

GFT: Google Flu Trends
RSV: Relative Search Volume
WHO: World Health Organization
