# Supplementary material for: Understanding Health Communication Through Google Trends and News Coverage for COVID-19: Multinational Study in Eight Countries
Source: JMIR Public Health Surveill. 2021 Dec 21;7(12):e26644. doi: 10.2196/26644 (PMC8691414; doi:10.2196/26644)
Supplement: Multimedia Appendix 1 [file publichealth_v7i12e26644_app1.docx]

**Multimedia Appendix 1:** Supplementary materials

In Figure S1, showed the time-lag correlation between the overall RSV for the topic “diseases” and daily new cases. We divided the 8 countries into 2 categories. The first category of countries included the United States, the United Kingdom, Canada, Ireland, and Singapore. Their maximum correlation appeared in the 17 days before the peak of daily new cases, and the maximum correlation is 0.9 (US) to 0.6 (Singapore). During the time lag of -17 and +17 days, the correlation gradually decreased, with an obvious negative linear trend, indicating that the public's interest in the topic of “Diseases” gradually decreased over time. Among them, in the United Kingdom, Singapore, and Ireland, negative correlations began to appear within 10-16 days after the peak of daily new cases. In the second category of countries (Australia, South Africa, and New Zealand), about 10, 5 days and 4 days before the peak of daily new cases, the public's interest in the topic of "Diseases" reached the highest level, with the maximum correlation degree exceeding 0.5. And the time lag correlation between -17 and 17 days showed a curve trend of middle high and low on both sides.

In Figure S2, showed the time-lag correlation between the overall RSV for the topic “treatments and medical resources” and daily new cases. Except for South Africa, the time-lag correlation between -17 days and +17 days in other countries showed significant negative correlation, and eight countries can be divided into four categories. In first category country (Singapore), within the time-lag of -17 and +17 days, the overall RSV of the "symptoms and signs" topic showed a significant negative correlation with the number of daily new cases. The degree of correlation appeared the maximum in 1 day before the peak of number of daily new cases, and showed a curve trend of high in the middle and low on both sides. For second category country (South Africa): The correlation was positive between the time-lag of -17 and +16 days, with the maximum correlation just over 0.4, and the degree of correlation decreased over time. The third category, which included the United States, United Kingdom, Canada, and Ireland, had the maximum correlation, more than 0.7, in the 17 days before the highest peak of daily new cases. They started to show negative correlation at -2 days, 6 days, -6 days and -2 days when the daily number of new cases peaked, respectively. The fourth category were Australia and New Zealand: During the 8 days before the peak of daily new cases, the correlation gradually increased, the maximum was close to 0.9, and then the correlation gradually decreased, and started to show negative correlation in 11 days after the peak number of daily new cases.

In Figure S3, except for Singapore, the overall RSV of the topic “public measure” in other countries was positively correlated with the number of daily new cases, with a maximum correlation coefficient reaching 0.9 (Australia). The first category of countries (Singapore): In 16 days before the peak of daily new cases, the public's interest in the topic “Public measures” reached the highest, but the maximum correlation was only over 0.4, and then the interest in the topic "Public measures" gradually decreased. And the negative correlation appeared in 10 days after the peak of daily new cases. The second category of countries (United States, United Kingdom, Canada, Ireland): Except for the United Kingdom, the maximum correlation appeared in the 17 days before the peak of daily new cases, and the maximum correlation was close to 0.8, then gradually decreased, indicating the public's interest in public measures gradually decrease over time. The third category of countries (Australia, South Africa, New Zealand): About 5 days, 1 day, and 2 days before the peak of daily new cases, the public's interest in the topic of "Public measures" reached the highest level, with the maximum correlation was close to 0.9. The time-lag correlation between -17 and 17 days showed a curve trend of middle high and low on both sides.

In Figure S4, except for Singapore, the overall RSV on the topic of “treatments and medical resources” had a positive correlation with the number of daily news in other countries, with the maximum correlation degree exceeding 0.8 (South Africa). The eight countries were divided into three categories, and the first category was Singapore. The correlation between the overall RSV of this topic and the number of daily news is low (less than 0.2), and it showed a negative correlation 6 days after the most news reports. That is, with the increase of total search volume of Treatments and medical resources, the number of daily news in Singapore showed a decreasing trend. The second category included the United States, Australia, and New Zealand, where public interest in the topic of Treatments and Medical Resources remained high during the first 17 days of maximum daily news coverage, with a peak correlation of nearly 0.8. However, two days after the peak of news coverage, one day before, and two days before, the correlation began to decrease, indicating that the public interest also gradually decreased. Countries in the third category, including the United Kingdom, Canada, Ireland, and South Africa, showed the highest interest in the topic “Treatments and medical resources” between -3 and +1 days, the daily news coverage formed peak, with a maximum correlation of over 0.8. In the time lag of -17 and 17 days, the correlation showed a curve trend of high in the middle and low on both sides.

In Figure S5, overall, the correlation between RSV on the topic of “symptoms and Signs” and the number of daily news was less than other topics, but the eight countries can still be divided into three categories. The first category is Singapore. Within the time lag of -17 and +17 days, the overall RSV of this topic presented a significant negative correlation with the number of daily news reports, and the correlation gradually increased with the change of time, indicating that with the increase of public interest or search queries on this topic, the number of news reports showed a decreasing trend. In the second categories (United Kingdom, Australia, South Africa, New Zealand, Canada, Ireland), there were a moderate level of interest in “Symptoms and Signs” for about 17 days before the peak of daily news coverage, with the maximum correlation above 0.6. However, there was a decrease in correlation for about 17 days after the peak of news coverage, suggesting that public interest was also decreasing. Among them, Canada and Ireland showed negative correlation after the maximum number of news reports was about 11 days and 14 days respectively. In the third category (the United States), the correlation was maximum in the 17 days before the peak of the number of daily news coverage, and then decreased gradually. And the correlation started to be negative about 4 days before the peak of news coverage, and gradually increased over time, with the maximum negative correlation exceeding -0.2.

In Figure S6, the overall RSV of the topic “public measures” in all countries has a positive correlation with the number of daily news, and the maximum correlation is close to 0.8. With the exception of the United States, the correlation between the overall RSV of the topic of “Public measures” and the number of daily news in other countries maintains a medium-to-high level, and eight countries were divided into three categories. In the first category (the United States), the maximum correlation appeared 17 days before the peak of daily news coverage, at nearly 0.8, and then the correlation declined in a very clear negative linear trend. In the second category (United Kingdom, Canada, Ireland, South Africa, New Zealand), interest in the Public Measures theme peaked 0-four days before its peak, with a correlation of nearly 0.8, and then began to decline. In the time lag of -17 and 17 days, the correlation showed a curve trend of high in the middle and low on both sides. In the third group of countries (Singapore, Australia), public interest in the topic was high during the 17 days before the maximum number of daily news reports, but the relevance gradually decreased after about 17 days of the maximum number of news reports.

**Table S1** showed the results of an interrupted time series analysis of the effect of the first covid-19 case on the search query of topic “symptoms and signs”. In the search queries of "shortness of breath", the occurrence of the first case caused a significant increase in the level of RSV in Ireland, but did not cause a significant change in the slope and level of RSV in other countries. For the search queries of "fever", the appearance of the first case caused a significant increase in the level of RSV in four countries (Ireland, Singapore, South Africa, and New Zealand). In addition, in South Africa, the first case also caused a significant increase in the slope of "fever" RSV. In the "cough" search queries, the announcement of the first case caused a significant increase in the level of RSV only in Ireland and New Zealand, but no significant changes in other countries. But for "tiredness", the announcement of the first case caused no significant change in the levels and slopes for eight countries.


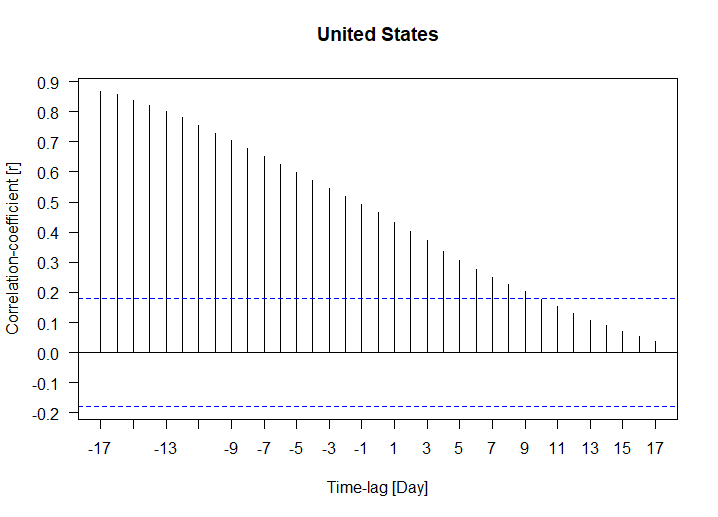

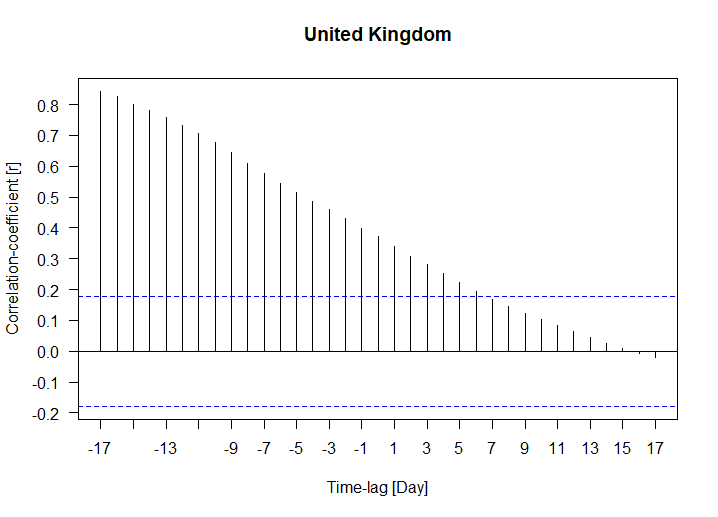


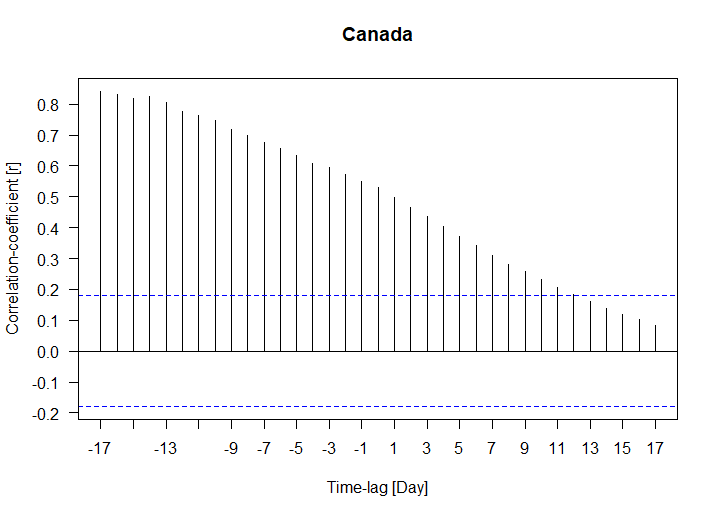

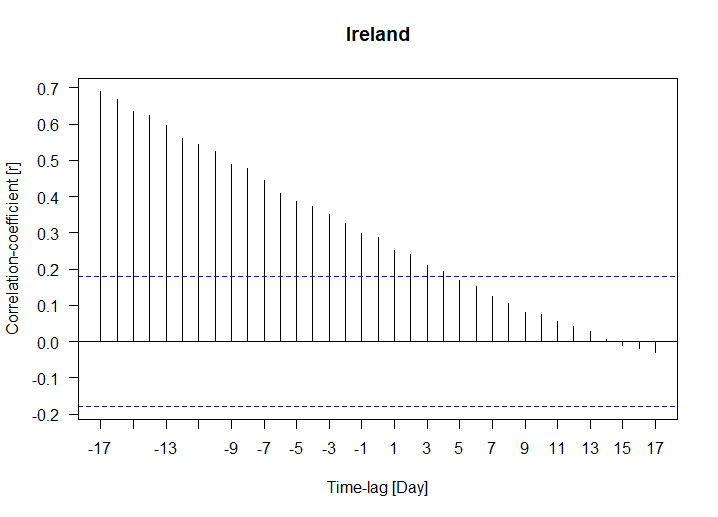


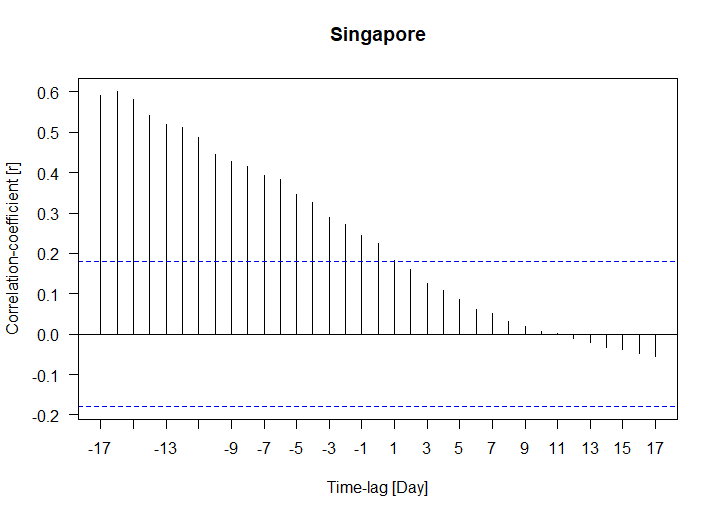

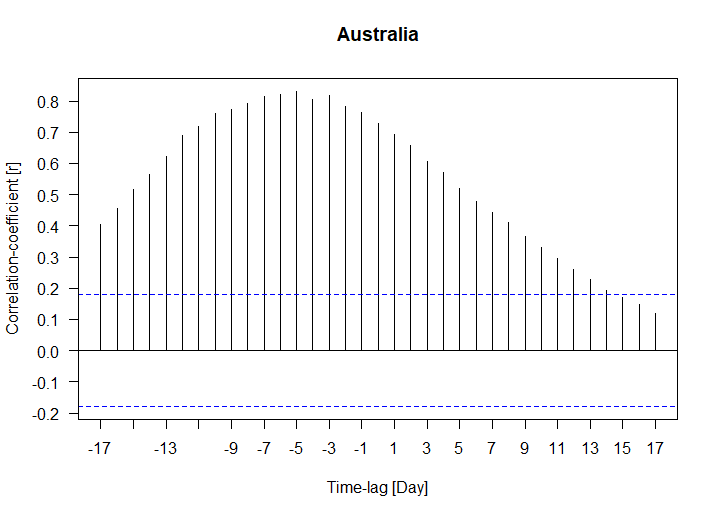


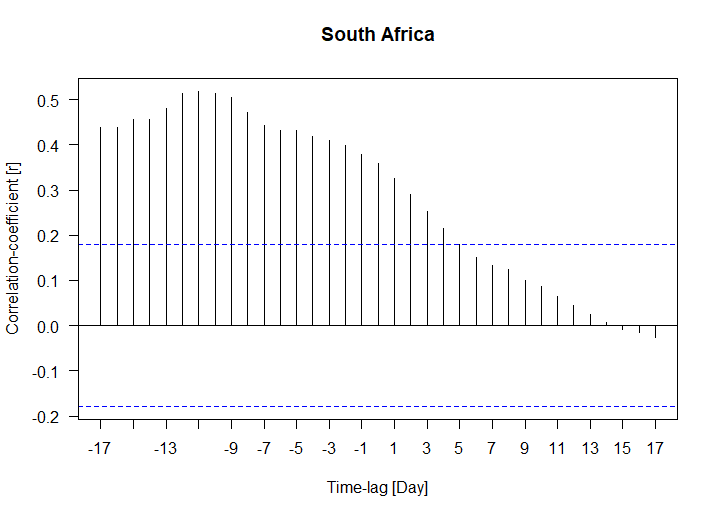

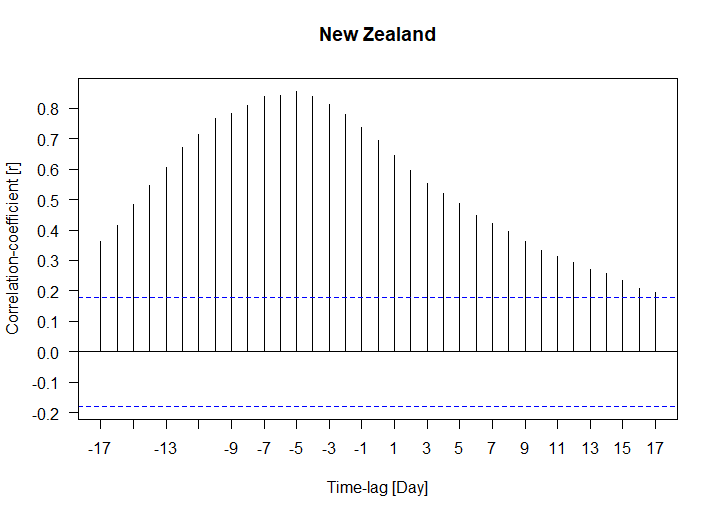


**Figure S1.** Time-lag correlations of the overall relative search volume (RSV) for “diseases” and new daily cases for eight countries from January 1, 2020, to April 29, 2020. The area between the two dotted blue lines is the 95% confidence interval of the white noise.


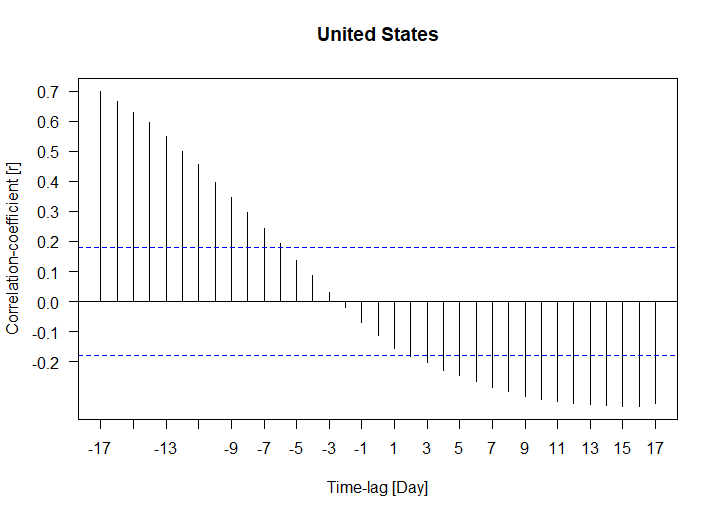

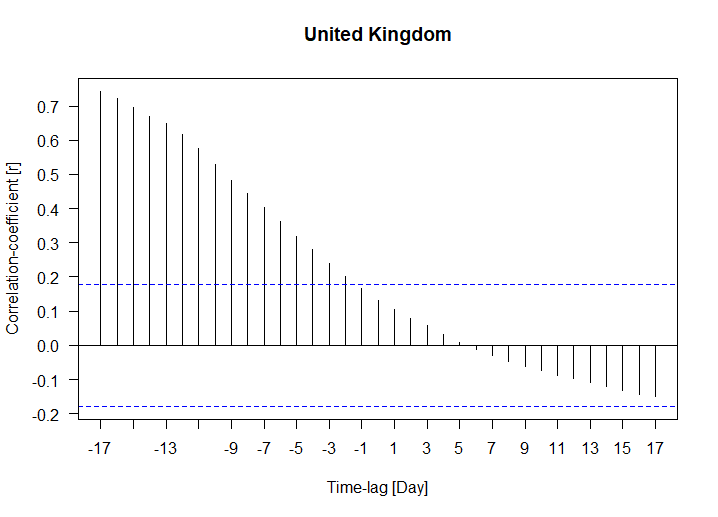


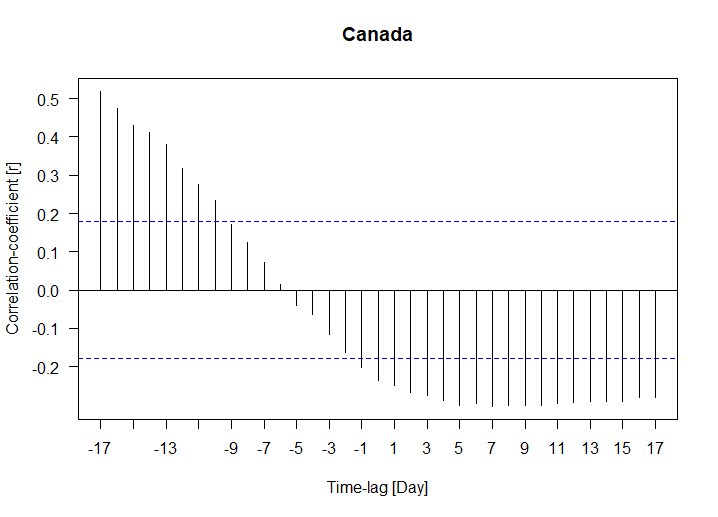

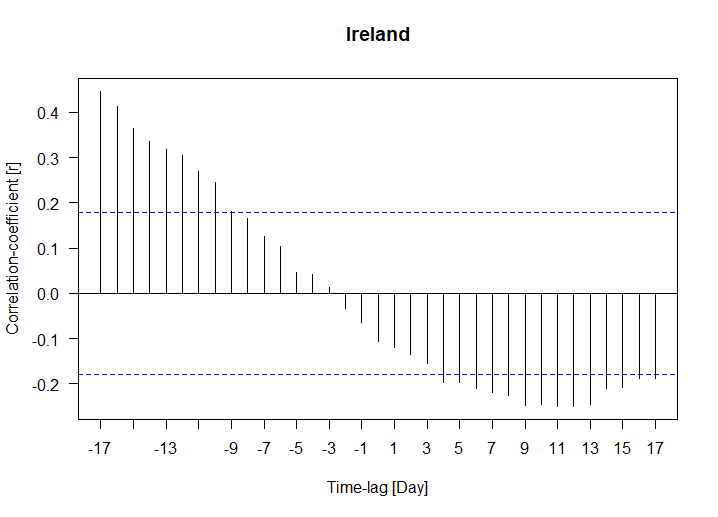


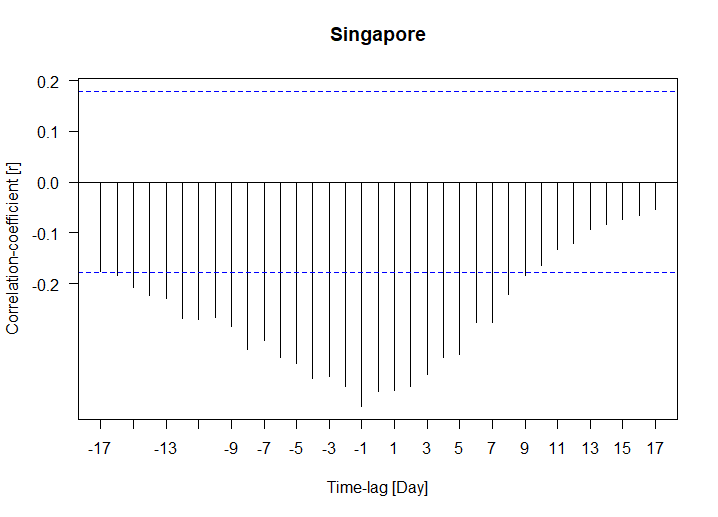

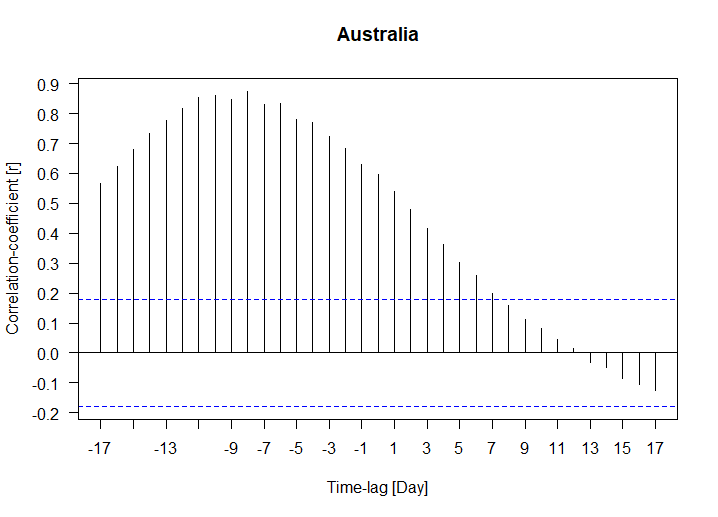


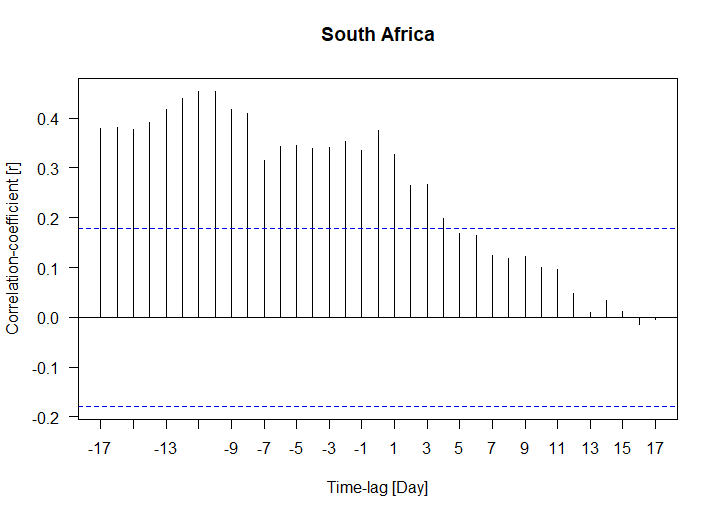

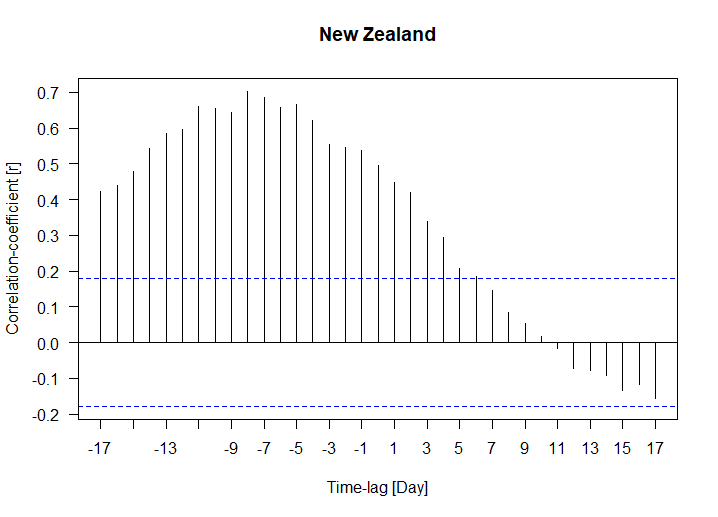


**Figure S2**. Time-lag correlations of the overall relative search volume (RSV) for “symptoms and signs” and new daily cases for eight countries from January 1, 2020, to April 29, 2020. The area between the two dotted blue lines is the 95% confidence interval of the white noise.


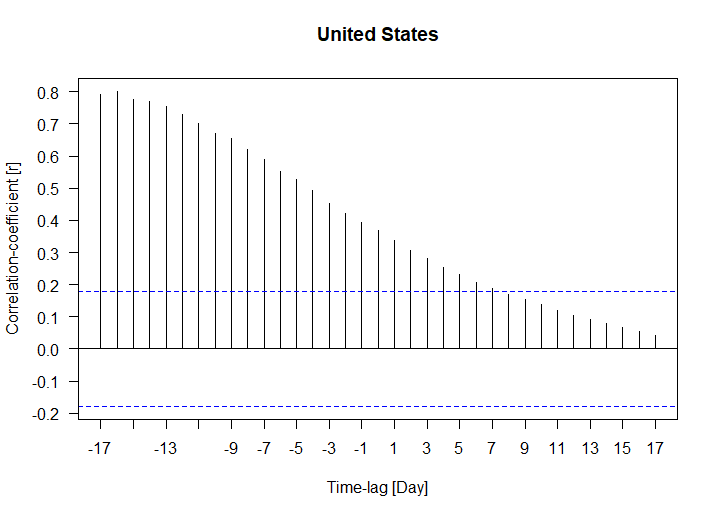

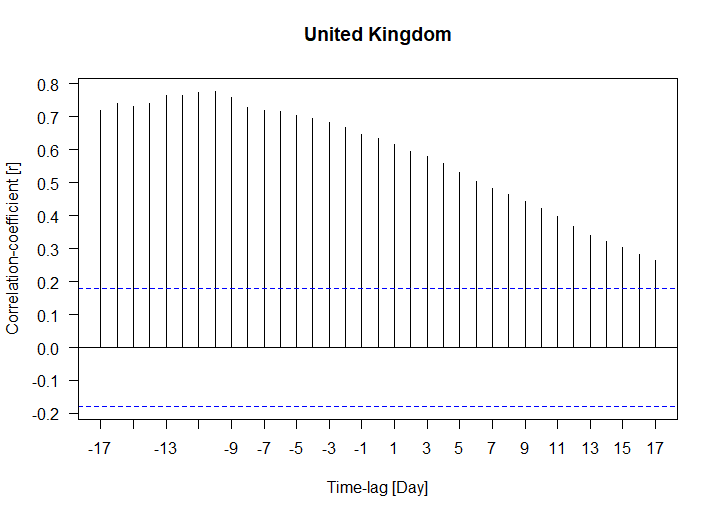


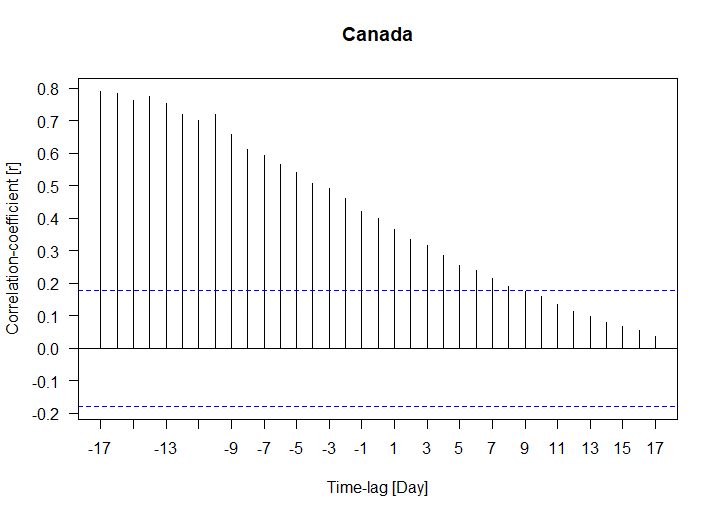

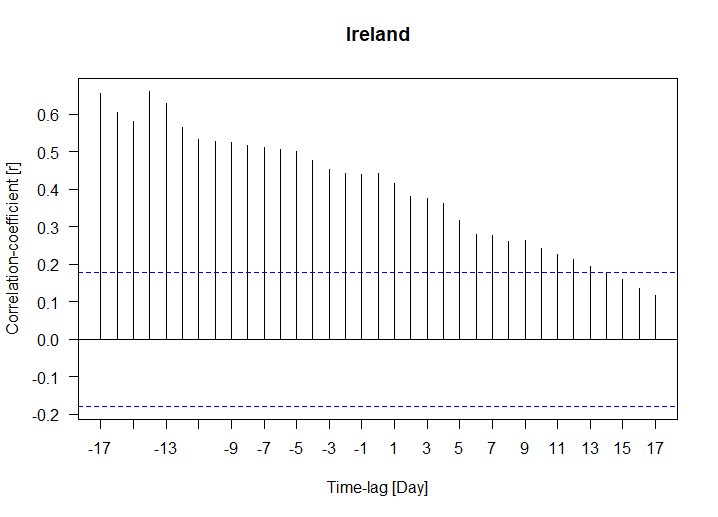


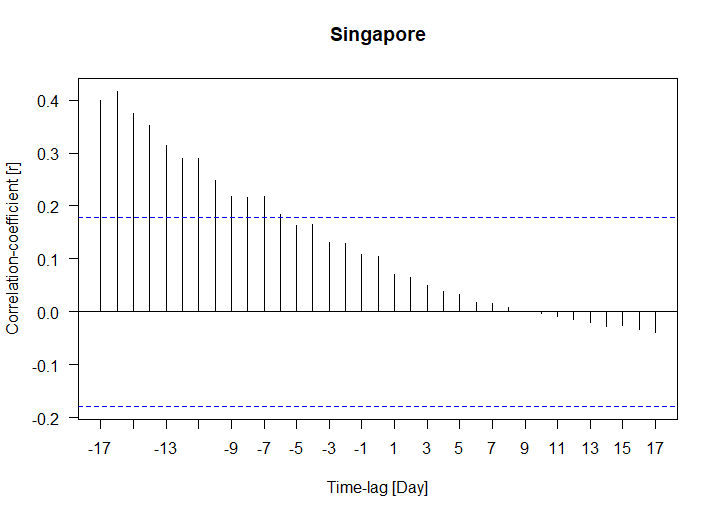

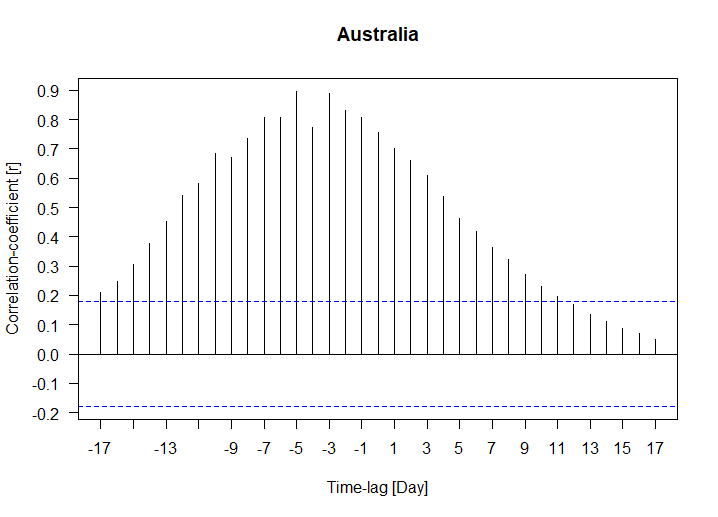


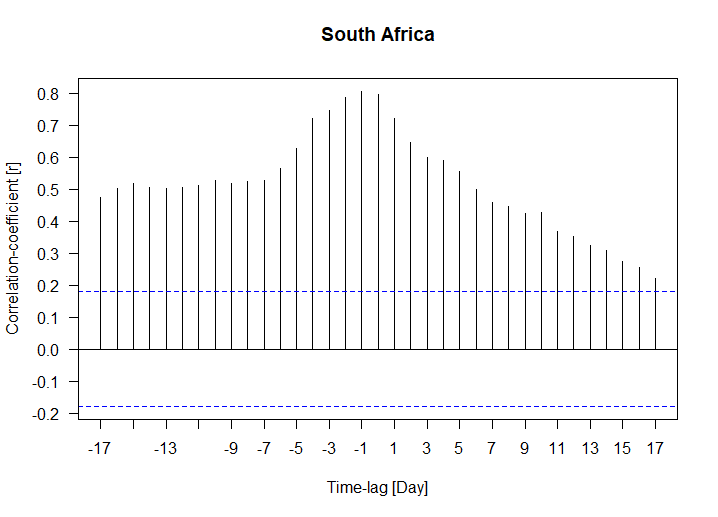

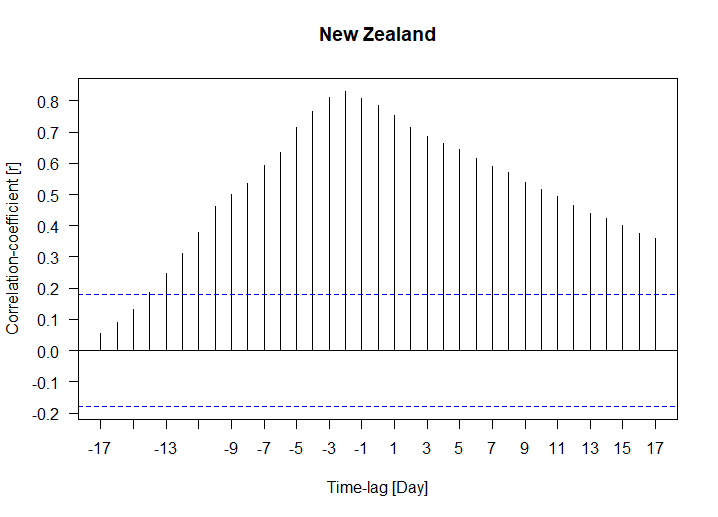


**Figure S3.** Time-lag correlations of the overall relative search volume (RSV) for “Public measures” and daily new cases for eight countries from January 1, 2020, to April 29, 2020. The area between the two dotted blue lines is the 95% confidence interval of the white noise.


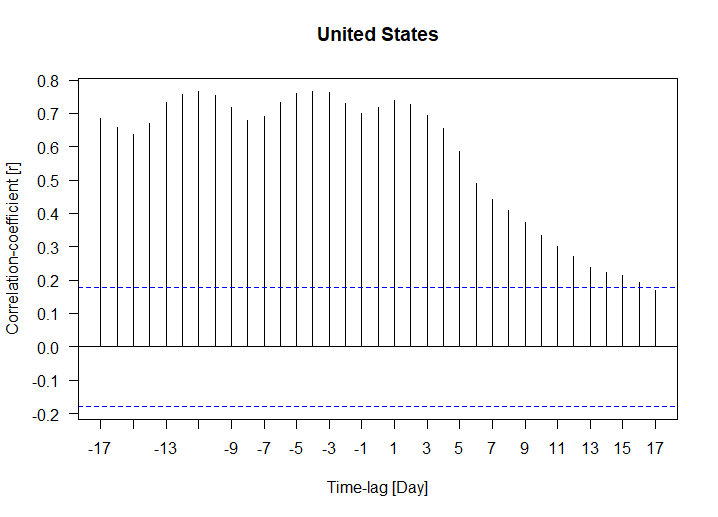

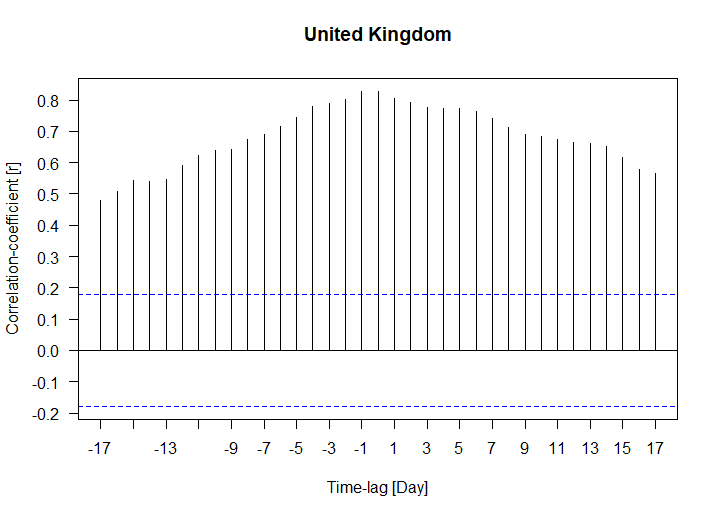


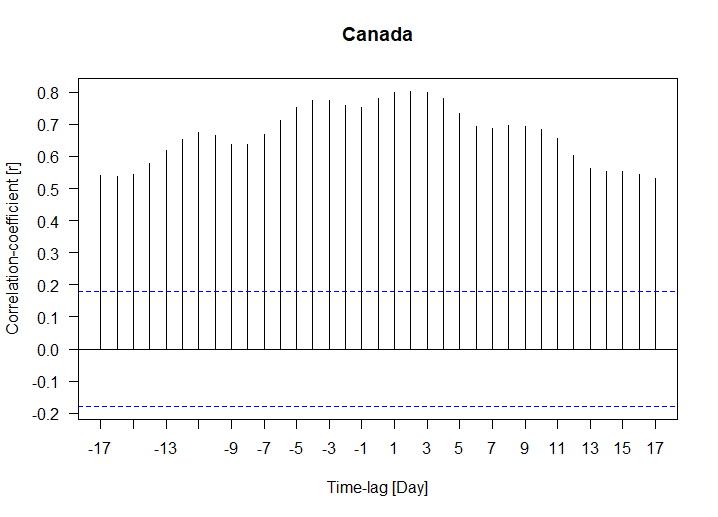

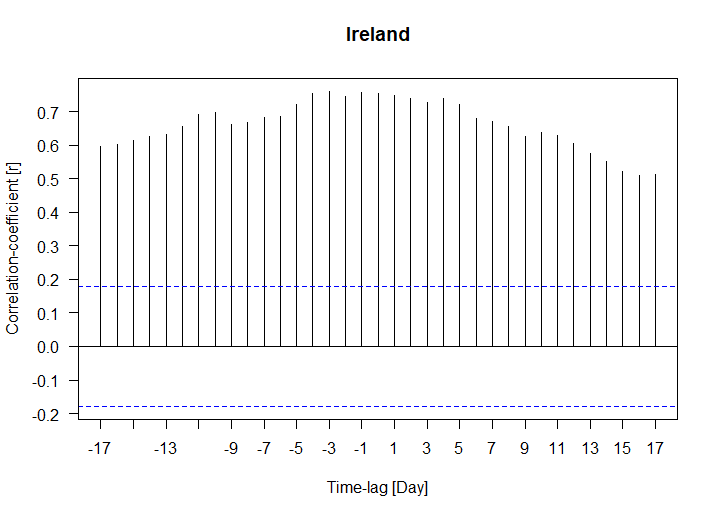


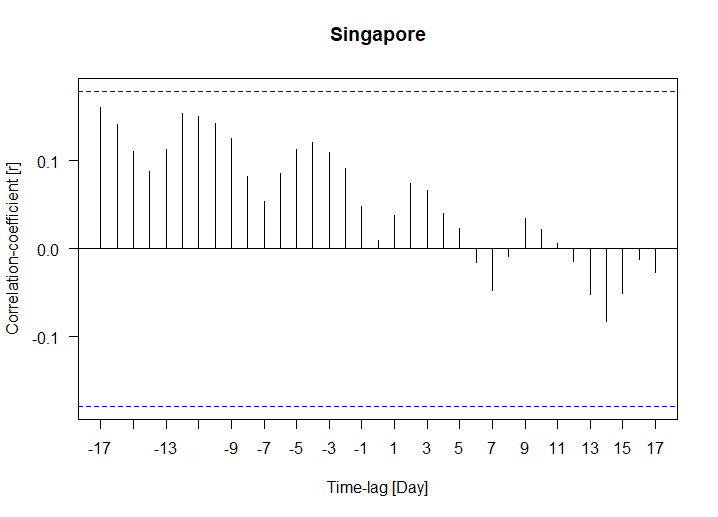

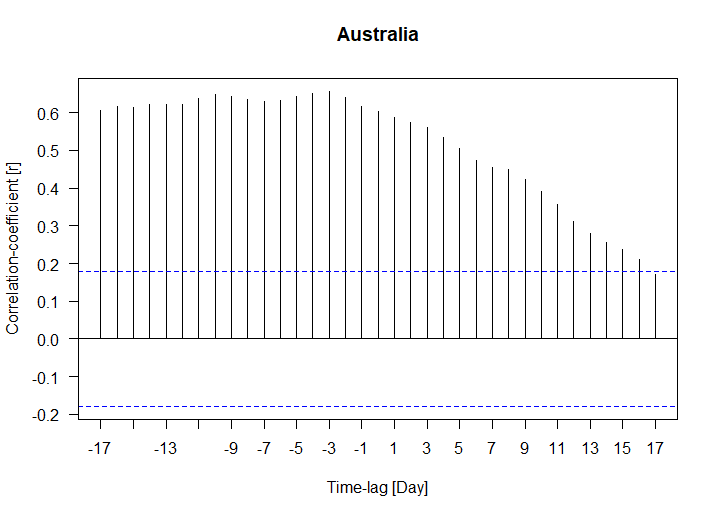


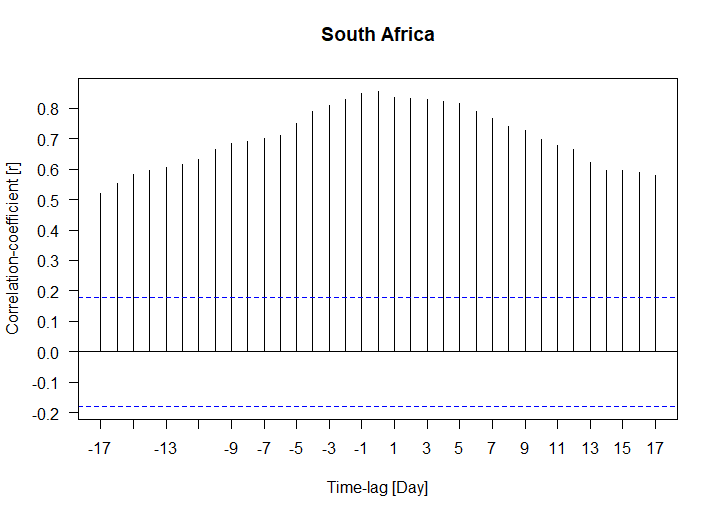

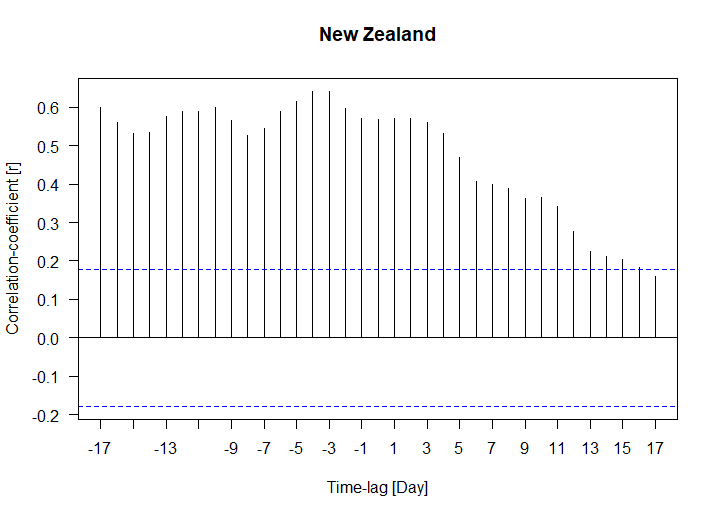
**Figure S4.** Time-lag correlations of the overall relative search volume (RSV) for “treatments and medical resources” and daily news items for eight countries from January 1, 2020, to April 29, 2020. The area between the two dotted blue lines is the 95% confidence interval of the white noise.


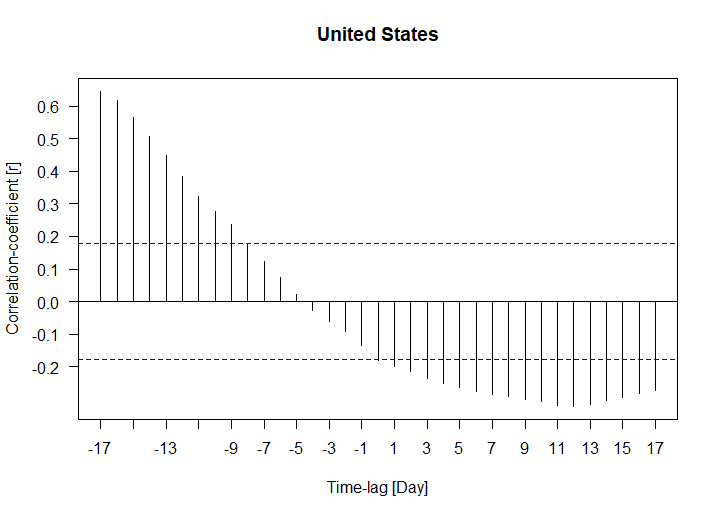

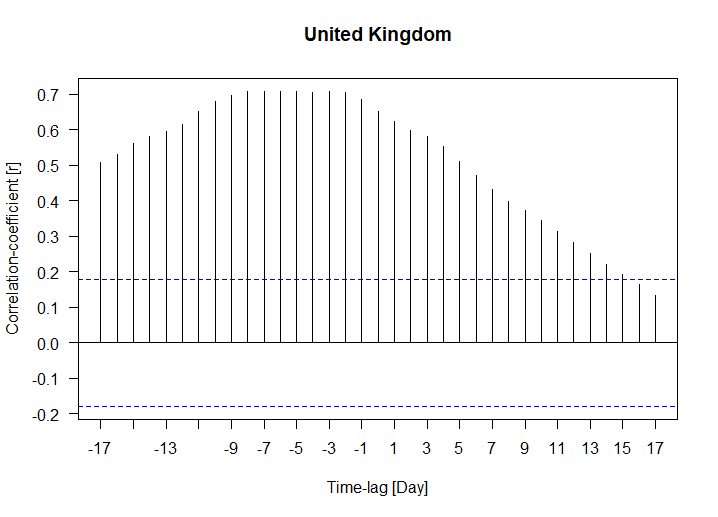


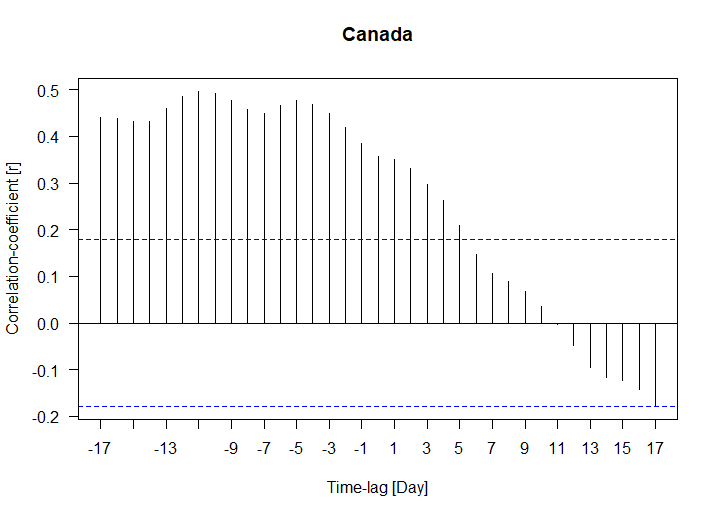

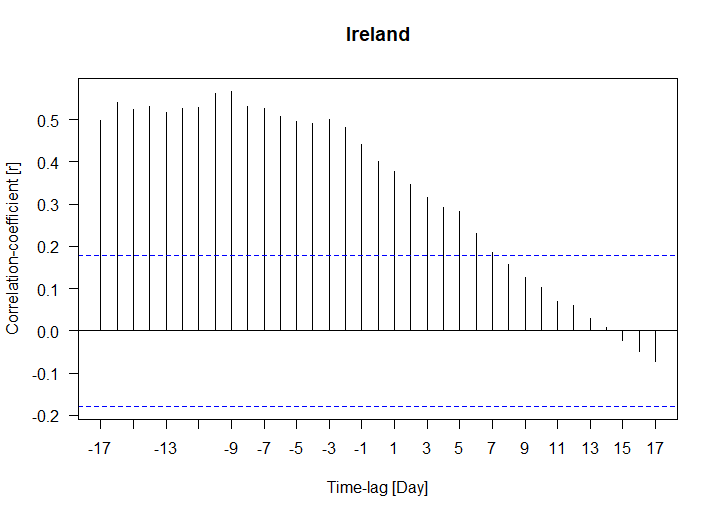


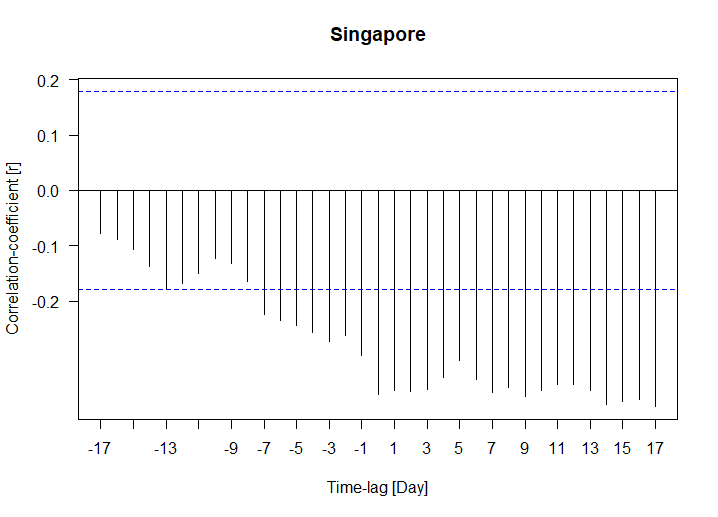

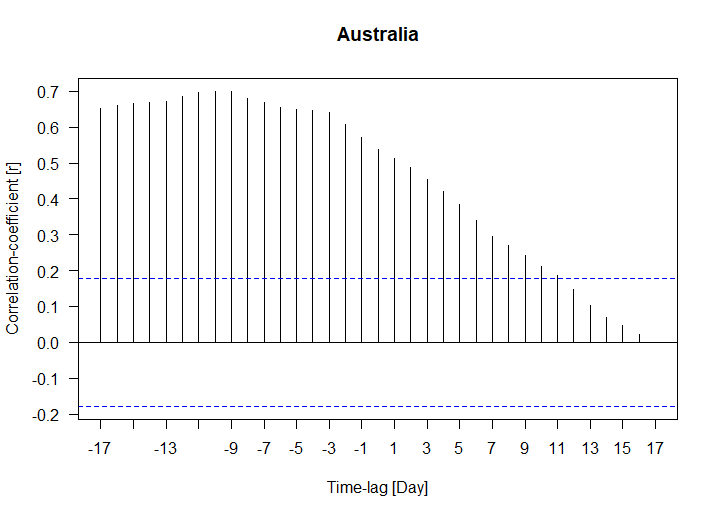


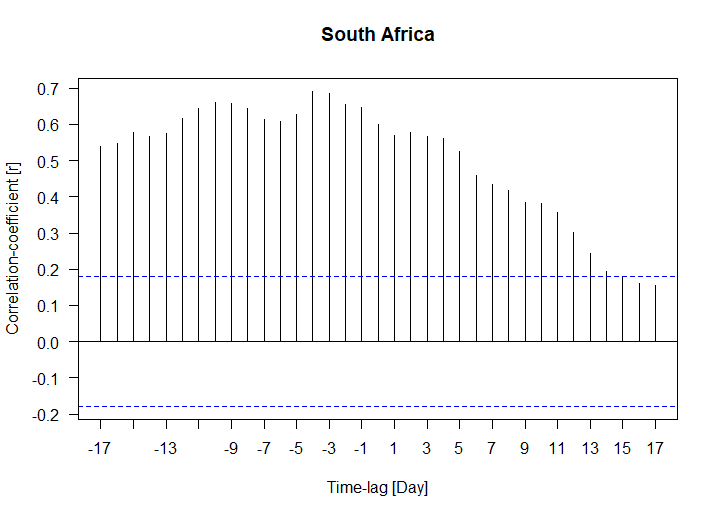

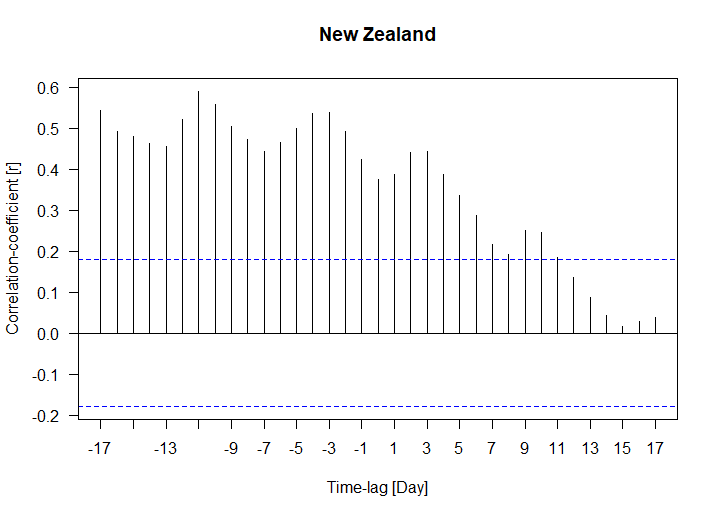


**Figure S5.** Time-lag correlations of the overall relative search volume (RSV) for “symptoms and signs” and daily news items for eight countries from January 1, 2020, to April 29, 2020. The area between the two dotted blue lines is the 95% confidence interval of the white noise.


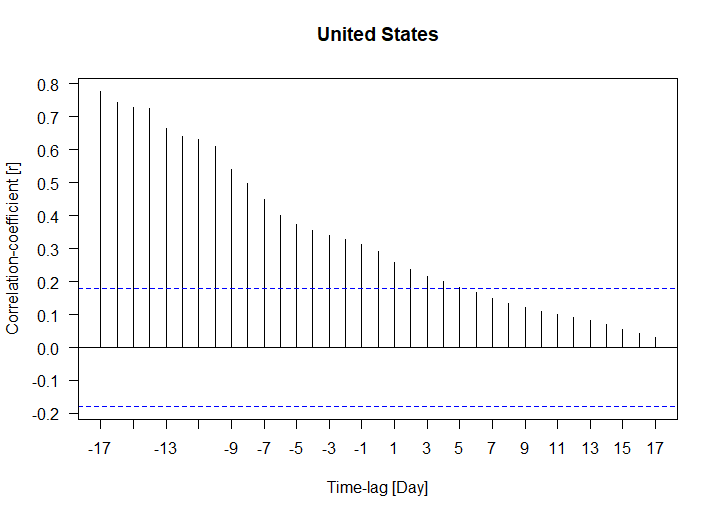

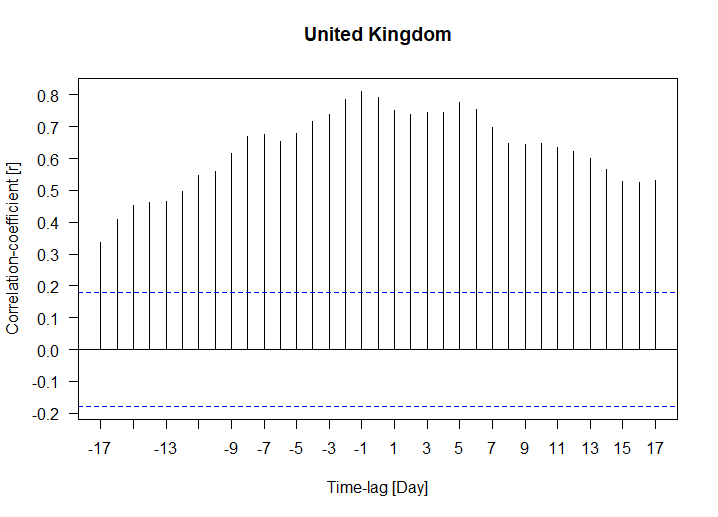


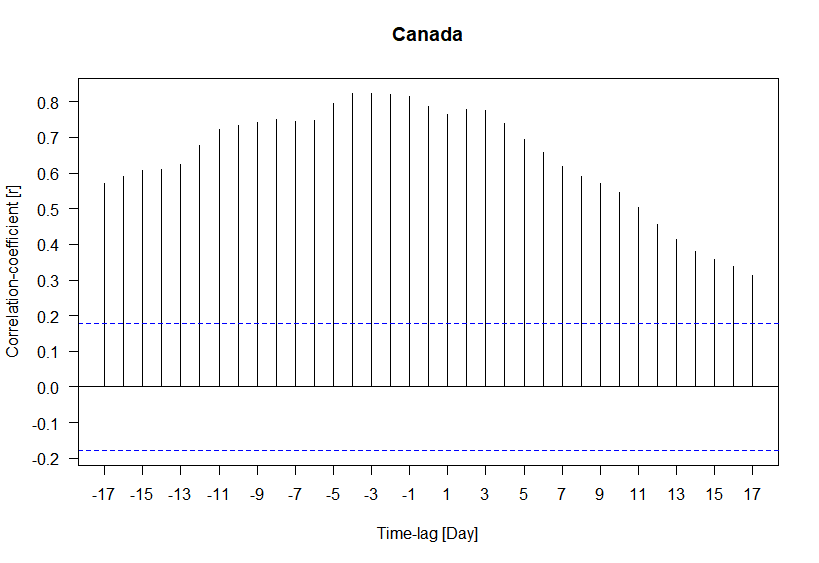

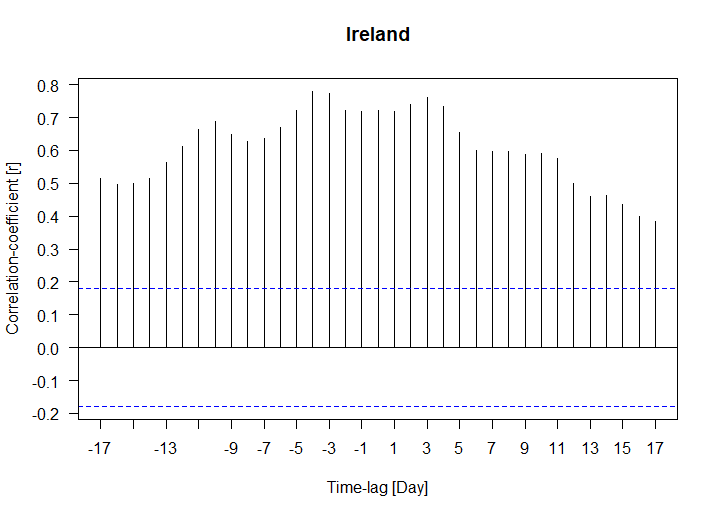


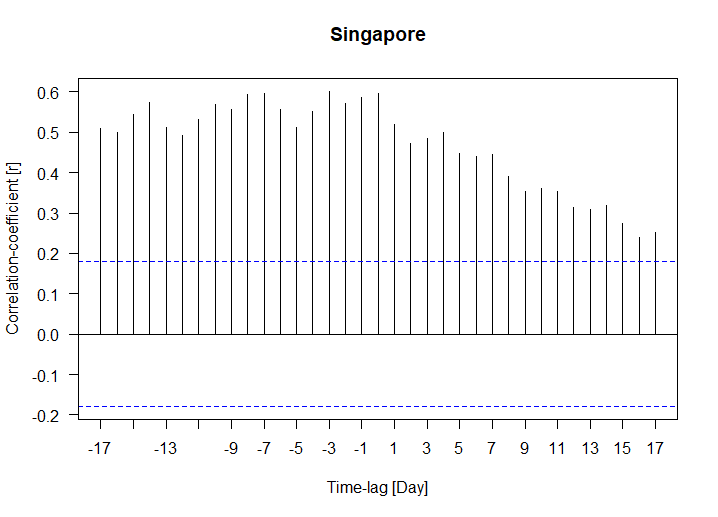

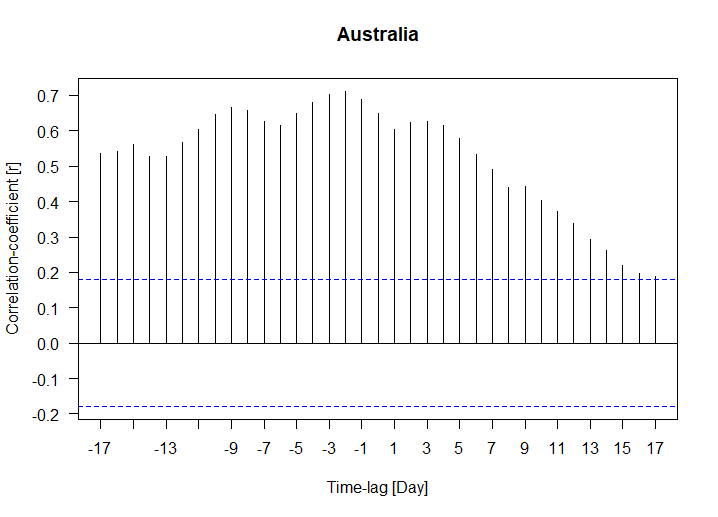


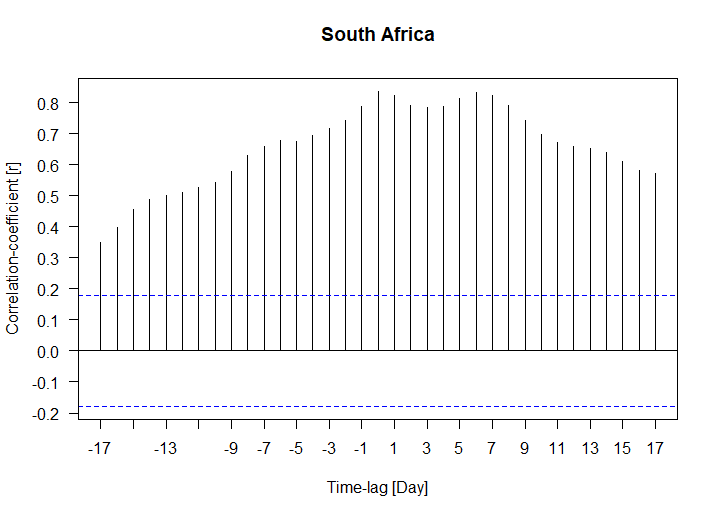

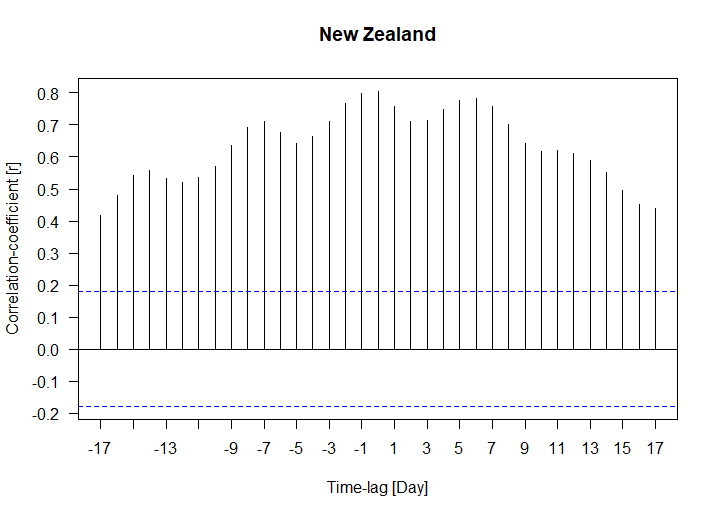


**Figure S6.** Time-lag correlations of the overall relative search volume (RSV) for “public measures” and daily news items for eight countries from January 1, 2020, to April 29, 2020. The area between the two dotted blue lines is the 95% confidence interval of the white noise.

**Table S1. Effect of the first COVID-19 case on the search queries (RSV) of the topic “symptoms and signs”.**

| **Country** | **Symptoms and signs** | **Changepoint date** | **Change in level** | | **Change in slope** | |
| --- | --- | --- | --- | --- | --- | --- |
|  |  |  | **Coefficient**  **(95% confidence interval)** | ***P-*value** | **Coefficient**  **(95% confidence interval)** | ***P-*value** |
| **United States** |  |  |  |  |  |  |
|  | Shortness of breath | Jan 20 | 0.03 (-1.94, 2.00) | 0.98 | 0.02 (-0.32, 0.35） | 0.92 |
|  | Fever | Jan 20 | -2.13 (-10.33, 6.07) | 0.61 | -0.34 (-2.23, 1.56) | 0.72 |
|  | Cough | Jan 20 | 0.63 (-5.92, 7.19) | 0.85 | 0.29 (-1.13, 1.71) | 0.69 |
|  | Tiredness | Jan 20 | 0.14 (-0.70, 0.36) | 0.19 | 0.01 (-0.01, 0.03) | 0.26 |
| **United Kingdom** |  |  |  |  |  |  |
|  | Shortness of breath | Feb 1 | 0.19 (-1.72, 1.59) | 0.79 | 0.01 (-0.09, 0.10) | 0.84 |
|  | Fever | Feb 1 | 1.13 (-6.09, 8.35) | 0.76 | -0.15 (-1.16, 0.87) | 0.77 |
|  | Cough | Feb 1 | 6.49 (-4.81, 17.78) | 0.26 | 0.17 (-1.50, 1.94) | 0.85 |
|  | Tiredness | Feb 1 | 0.47 (-0.13, 1.06) | 0.13 | 0.00 (-0.03, 0.03) | 0.79 |
| **Canada** |  |  |  |  |  |  |
|  | Shortness of breath | Jan 26 | 1.30 (-1.00, 3.59) | 0.27 | 0.05 (-0.10, 0.20) | 0.49 |
|  | Fever | Jan 26 | -10.00 (-27.19, 7.19) | 0.25 | -0.81 (-2.82, 1.20) | 0.43 |
|  | Cough | Jan 26 | -6.09 (-16.89, 4.71) | 0.27 | -0.16 (-1.24, 0.92) | 0.77 |
|  | Tiredness | Jan 26 | -0.37 (-0.94, 0.20) | 0.20 | -0.02 (-0.06, 0.01) | 0.17 |
| **Ireland** |  |  |  |  |  |  |
|  | Shortness of breath | Mar 1 | 4.32 (1.16, 7.47) | 0.01 | -0.07 (-0.16, 0.03) | 0.17 |
|  | Fever | Mar 1 | 19.91 (7.55, 32.26) | 0.00 | -0.37 (-0.75, 0.01) | 0.06 |
|  | Cough | Mar 1 | 32.22 (15.22, 49.22) | 0.00 | -0.49 (-1.06, 0.08) | 0.09 |
|  | Tiredness | Mar 1 | -0.10 (-2.00, 1.79) | 0.92 | -0.01 (-0.06, 0.04) | 0.72 |

**Table S1. (continued).**

| **Country** | **Symptoms and signs** | **Changepoint date** | **Chang in level** | | **Change in slope** | |
| --- | --- | --- | --- | --- | --- | --- |
|  |  |  | **Coefficient**  **(95% confidence interval)** | ***P-*value** | **Coefficient**  **(95% confidence interval)** | ***P-*value** |
| **Singapore** |  |  |  |  |  |  |
|  | Shortness of breath | Jan 24 | 2.37 (-0.59,5.33) | 0.16 | 0.07 (-0.13, 0.27) | 0.48 |
|  | Fever | Jan 24 | 25.26 (7.32, 43.21) | 0.01 | 0.24 (-0.97, 1.44) | 0.70 |
|  | Cough | Jan 24 | 5.74 (-3.79, 15.26) | 0.24 | -0.46 (-1.09, 0.17) | 0.15 |
|  | Tiredness | Jan 24 | -0.35 (-1.61, 0.91) | 0.58 | -0.02 (-0.11, 0.66) | 0.58 |
| **Australia** |  |  |  |  |  |  |
|  | Shortness of breath | Jan 25 | 0.27 (-1.86, 2.40) | 0.80 | 0.03 (-0.10, 0.17) | 0.63 |
|  | Fever | Jan 25 | 8.55 (-9.44, 26.55) | 0.35 | 0.11 (-1.45,1.66) | 0.89 |
|  | Cough | Jan 25 | 4.40 (-8.84, 17.64) | 0.51 | -0.12 (-1.40, 1.16) | 0.85 |
|  | Tiredness | Jan 25 | 0.39 (-0.79, 1.57) | 0.52 | -0.00 (-0.08, 0.07) | 0.96 |
| **South Africa** |  |  |  |  |  |  |
|  | Shortness of breath | Mar 5 | 3.06 (0.24, 5.88) | 0.82 | -0.01 (-0.09, 0.07) | 0.81 |
|  | Fever | Mar 5 | 26.81 (16.95, 36.66) | 0.00 | -0.56 (-0.85, -0.27) | 0.00 |
|  | Cough | Mar 5 | 3.51 (-7.53, 14.54) | 0.53 | -0.21 (-0.65, 0.23) | 0.35 |
|  | Tiredness | Mar 5 | -0.05(-1.75, 1.65) | 0.96 | -0.02 (-0.70, 0.03) | 0.44 |
| **New Zealand** |  |  |  |  |  |  |
|  | Shortness of breath | Feb 28 | 3.43 (-0.63, 7.50) | 0.10 | 0.03 (-0.09, 0.07) | 0.64 |
|  | Fever | Feb 28 | 17.16 (2.87, 31.46) | 0.02 | -0.31 (-0.73, 0.10) | 0.14 |
|  | Cough | Feb 28 | 28.06 (14.93, 41.18) | 0.00 | -0.38 (-0.76, 0.01) | 0.05 |
|  | Tiredness | Feb 28 | -0.83 (-3.82, 2.17) | 0.59 | -0.02 (-0.11, 0.07) | 0.63 |
